# Nationwide Trends in Screen Time and Associated Risk Factors by Family Structures Among Adolescents, 2008-2022: Nationwide Cross-Sectional Study

**DOI:** 10.2196/57962

**Published:** 2025-03-10

**Authors:** Seokjun Kim, Hyesu Jo, Yejun Son, Min Kyung Shin, Kyeongmin Lee, Jaeyu Park, Hayeon Lee, Lee Smith, Elena Dragioti, Guillaume Fond, Laurent Boyer, Guillermo F López Sánchez, Mark A Tully, Masoud Rahmati, Damiano Pizzol, Selin Woo, Dong Keon Yon

**Affiliations:** 1 Department of Medicine Kyung Hee University College of Medicine Seoul Republic of Korea; 2 Center for Digital Health, Medical Science Research Institute Kyung Hee University College of Medicine Seoul Republic of Korea; 3 Department of Regulatory Science Kyung Hee University Seoul Republic of Korea; 4 Department of Precision Medicine Kyung Hee University College of Medicine Seoul Republic of Korea; 5 Department of Medicine Wonkwang University College of Medicine Iksan Republic of Korea; 6 Department of Electronics and Information Convergence Engineering Kyung Hee University Yongin Republic of Korea; 7 Centre for Health, Performance and Wellbeing Anglia Ruskin University Cambridge United Kingdom; 8 Research Laboratory Psychology of Patients, Families, and Health Professionals, Department of Nursing, School of Health Sciences University of Ioannina Ioannina Greece; 9 CEReSS-Health Service Research and Quality of Life Center, Assistance Publique-Hôpitaux de Marseille Aix-Marseille University Marseille France; 10 Division of Preventive Medicine and Public Health, Department of Public Health Sciences, School of Medicine University of Murcia Murcia Spain; 11 School of Medicine Ulster University Londonderry United Kingdom; 12 Department of Physical Education and Sport Sciences, Faculty of Literature and Human Sciences Lorestan University Khoramabad Iran; 13 Department of Physical Education and Sport Sciences, Faculty of Literature and Humanities Vali-E-Asr University of Rafsanjan Rafsanjan Iran; 14 Health Unit Eni San Donato Milanese Italy; 15 Health Unit Eni Maputo Mozambique; 16 Department of Pediatrics, Kyung Hee University Medical Center Kyung Hee University College of Medicine Seoul Republic of Korea

**Keywords:** adolescents, family type, pandemic, screen time, South Korea, sedentary activity, risk factor, mobile phone

## Abstract

**Background:**

Although understanding long-term trends in adolescent screen time and the influence of family structure is essential, there is a lack of research addressing these issues comprehensively.

**Objective:**

This study aimed to conduct comprehensive investigations into adolescent screen time before and during the COVID-19 pandemic, with a particular focus on family structures.

**Methods:**

This study used nationwide, large-scale data from the Korea Youth Risk Behavior Web-Based Survey from South Korea. We aimed to indicate the changes in adolescent screen time over 15 years from 2008 to 2022. Weighted linear regression was used to analyze annual trends in screen time before and during the pandemic, and stratified analyses were conducted to examine associated risk factors across different family structures.

**Results:**

This study used data from a total of 836,972 individuals (n=403,456, 48.2% women), with an age range of 12-18 years. The analysis revealed an overall increase in screen time prepandemic (β=8.06, 95% CI 7.74-8.39), with a notable increase observed at the onset of the pandemic (β=162.06, 95% CI 159.49-164.64). Among diverse family structures, the orphanage group showed the most substantial increase in screen time during the pandemic (β_diff_=221.90, 95% CI 159.62-284.17). Risk factors associated with screen time during the pandemic varied by family structure. Notably, the nuclear family group presented distinct screen time–related risk factors, including grade, region of residence, physical activity frequency, sadness and despair, and the highest education level of parents.

**Conclusions:**

There has been a notable increase in average screen time among adolescents since the onset of the pandemic, with the orphanage group exhibiting a pronounced trend. The risk factors associated with screen time during the pandemic varied for each family structure. Findings from this study suggest that the implementation of individualized measures tailored to each family structure should be adopted to effectively address the increased issue of adolescent screen time since the pandemic.

## Introduction

There was a declaration of COVID-19 as a global pandemic by the World Health Organization in March 2020 [1]. With the proclamation, countries worldwide have advocated for indoor living, remote work, and web-based learning to prevent the spread of COVID-19. For example, the National Health Service in England advised individuals to remain at home and participate in remote work unless necessary, while the Chinese government implemented restrictions on public transport and postponed the reopening of schools following the vacation period [2]. Globally, universities and educational institutions were closed, prompting a rapid digital transition in the education system [3,4]. The increase in indoor time since the pandemic has likely induced a concurrent increase in screen time [5,6]. Throughout the pandemic, social distancing and economic hardships restricted daily activities and social interactions, negatively impacting individuals’ mental health worldwide [7,8]. Among preschool children, psychological resilience has been identified as a factor that amplifies the positive outcomes associated with the use of digital technologies [9]. The need for discussing how the utilization of digital technologies evolves in response to mental health changes brought about by the pandemic is increasingly evident. In such circumstances, specific focus is placed on the increased screen time of adolescents, due to the association with adolescent depression and mental health [10,11]. Global guidelines, such as Canada’s 24-Hour Movement Guidelines for Children and Youth, recommend limiting recreational screen time to 2 hours or less per day, highlighting the potential risks of excessive screen use [12]. With the onset of the COVID-19 pandemic, there is growing global attention to screen time among adolescents.

In South Korea, concerns about excessive screen time among adolescents predate the pandemic. The “Shutdown Law,” implemented in 2011, restricted web-based gaming for those younger than 16 years old during late-night hours to prevent gaming addiction but was abolished in 2021 [13]. A recent study highlighted a significant increase in screen time among Korean adolescents, with teenagers spending an average of 303.66 minutes daily during the pandemic, a substantial rise from 133.24 minutes in the prepandemic period [14]. The screen time of adolescents appears to be influenced by their family structure, likely due to variations in parental monitoring, restrictions on screen use, and the promotion of outdoor activities [15]. However, studies on this topic are limited by small sample sizes, a narrow focus on specific family structures, and a lack of consideration for the significant changes brought about by the COVID-19 pandemic [15,16]. This underscores the need for a comprehensive analysis of long-term trends in adolescent screen time, particularly about diverse family structures, both before and during the pandemic.

Therefore, this study investigated the 15-year trends in adolescent screen time and potential risk factors based on family structures, using a nationwide dataset of Korean adolescents surveyed between 2008 and 2022. The primary aim of this study was to assess the changes in adolescent screen time trends and associated risk factors in each family structure since the onset of the pandemic.

## Methods

### Study Design and Population

This study used the Korea Youth Risk Behavior Web-Based Survey (KYRBS). KYRBS is a large-scale, nationwide survey covering health-related behaviors of Korean adolescents [17]. The KYRBS is designed to collect data from middle and high school students (grades 7 to 12) aged 12 to 18 years enrolled in public and private schools throughout South Korea. To achieve national representativeness, the survey uses a stratified, multistage cluster sampling approach [18]. Schools are chosen as the primary sampling units based on regional and school-type stratifications, followed by a random selection of classes within these schools [18]. This methodology ensures a balanced representation of students from both urban and rural areas. Administered annually by the Korea Disease Control and Prevention Agency, the survey has consistently maintained an average response rate of 95.7% over the past 15 years. Students voluntarily completed a web-based anonymous questionnaire following a standardized protocol in a computer lab in each school [19].

To ensure the data’s representativeness, sampling weights were determined and applied. The weighting process considers the probability of selection at each stage of sampling (schools and classes), adjusts for nonresponse, and incorporates poststratification adjustments to align with the overall student population distributions by grade, sex, and school type for each survey year. To avoid overrepresentation, extreme weights were capped using interquartile range techniques. These adjustments ensure that the results reliably represent the health behaviors of the adolescent population across South Korea. In this study, an initial cohort of 992,702 respondents completed the questionnaires from 2008 to 2022. Following the exclusion of participants with missing data, the final dataset comprised 836,972 samples (84.3% of the initial raw data). The exclusion of missing data was conducted systematically to maintain dataset robustness and minimize bias.

### Ethical Considerations

The study protocol was approved by the Institutional Review Board of the Korea Disease Control and Prevention Agency (2014-06EXP-02-P-A), and all participants provided written informed consent. Additionally, the study complied with the Population Health Promotion Act 19 (117058) as mandated by the Korean government. This study was conducted following the principles of the Declaration of Helsinki.

### Endpoints

We aimed to investigate trends in screen time and explore associated risk factors, with a focus on the diverse family structures of adolescents. The COVID-19 pandemic period was defined as 2020-2022, starting with the first observed COVID-19 case in South Korea on January 20, 2020, and aligning with widespread public health measures [20,21]. The prepandemic years were grouped into 6-year intervals, each spanning two consecutive years, as follows: 2008-2009, 2010-2011, 2012-2013, 2014-2015, 2016-2017, and 2018-2019. This classification allows for a clear comparison of trends before and during the pandemic.

Screen time was defined as the average daily time spent on the internet or using smartphones for noneducational purposes. Participants were asked: “What was your average daily internet or smartphone use on weekdays in the past 30 days?” and “What was your average daily internet or smartphone use on weekends in the past 30 days?” The overall average screen time was calculated by combining weekday and weekend use, weighted to reflect their relative proportions within a week [19].

Family structures were categorized into 4 groups: nuclear family, living with relatives, living alone, and orphanage. A nuclear family was defined as adolescents living with one or both parents, regardless of biological relationships. The “living with relatives” category included adolescents residing with extended family members, such as grandparents or uncles or aunts, excluding parents. “Living alone” referred to those managing households independently without the presence of any family members. The “orphanage” category encompassed adolescents living in child welfare facilities. These classifications are based on established definitions in prior studies on family dynamics and adolescent health [22].

### Covariate Definitions

A total of 11 covariates were included in the analytical models: sex, BMI group (underweight, normal, overweight, and obese), grade (7th to 9th [middle-school] and 10th to 12th [high-school]), region of residence (urban and rural) [23,24], smoking status, alcoholic consumption, school performance (low, middle-low, middle, middle-high, and high), sexual experience, physical activity frequency (lower activity, moderate activity, and higher activity), sadness and despair, and highest education level of parents (high school or lower and college or higher). BMI was calculated using self-reported height and weight data. Conforming to the 2017 Korean National Growth Charts, BMI was categorized into the following four groups: underweight (<5%), normal (5% to 84 %), overweight (85% to 94%), and obese (≥95%) [19]. School performance was categorized into the following five groups according to the self-reports of students: low (<20%), middle-low (20% to 39%), middle (40% to 59%), middle-high (60% to 79%), and high (≥80%). Physical activity frequency was classified into the following three groups based on the engagement in vigorous aerobic and resistance training more than three days per week: lower activity (neither activity is done for more than three days per week), moderate activity (either one activity), and higher activity (both activities). The definitions of variables were extracted from peer-reviewed literature [19].

### Statistical Analysis

We used a weighted linear regression model to compute β coefficient and β difference (β_diff_) along with a 95% CI [25]. This strategy was selected for trend analysis and associated factor analysis. In the analysis of screen time trends, the β coefficient expressed the annual trend of screen time among adolescents in prepandemic or pandemic. β_diff_ was used to indicate the trend difference between the divided year groups based on the onset of the pandemic. In the analysis of screen time-associated factors, the β coefficient represents the correlation between screen time and each biological, social, and familial factor. β_diff_ was also used to reveal the difference in correlation between the year groups divided by the onset of the pandemic. In addition, the associations between screen time and each variable were recalculated after adjusting for potential influencing factors, including age, sex, BMI group, grade, region of residence, smoking status, alcoholic consumption, school performance, sexual experience, physical activity frequency, sadness and despair, and highest education level of parents. Statistical significance, as applied in all the above analyses, was determined by a 2-sided *P* value less than .05. Statistical analysis in this study was conducted using SAS software (version 9.4; SAS Institute Inc).

## Results

This study was conducted using KYRBS spanning from 2008 to 2022. A total of 836,972 individuals (n=403,456, 48.20% women) with an average age of 15.01 (SD 1.75) years were finally selected for the analysis following the missing data handling. Baseline characteristics of the raw data are indicated in Table 1.

**Table 1:** General characteristics of participants in KYRBS^a^, 2008-2022 (n=836,972).

		Total	Prepandemic							Pandemic
			2008-2009	2010-2011	2012-2013	2014-2015	2016-2017	2018-2019	2020-2022	
Overall, n (%)		836,972 (100)	118,457 (14.15)	121,079 (14.47)	111,432 (13.31)	109,654 (13.1)	115,169 (13.76)	101,771 (12.16)	159,410 (19.05)	
Age (years), mean (SD)		15.01 (1.75)	15.03 (1.75)	15.08 (1.75)	14.90 (1.76)	14.95 (1.75)	15.00 (1.74)	15.00 (1.78)	15.10 (1.74)	
**Sex, n (%)**										
	Men	433,516 (51.8)	63,372 (53.5)	63,168 (52.17)	58,694 (52.67)	56,442 (51.47)	58,685 (50.96)	51,467 (50.57)	81,688 (51.24)	
	Women	403,456 (48.2)	55,085 (46.5)	57,911 (47.83)	52,738 (47.33)	53,212 (48.53)	56,484 (49.04)	50,304 (49.43)	77,722 (48.76)	
**BMI group^b^, n (%)**										
	Underweight	65,467 (7.82)	10,586 (8.94)	10,485 (8.66)	8649 (7.76)	8390 (7.65)	7972 (6.92)	6850 (6.73)	12,535 (7.86)	
	Normal	608,013 (72.64)	89,246 (75.34)	91,583 (75.64)	83,935 (75.32)	81,264 (74.11)	82,875 (71.96)	71,846 (70.6)	107,264 (67.29)	
	Overweight	68,622 (8.2)	8068 (6.81)	8428 (6.96)	8496 (7.62)	8849 (8.07)	10,046 (8.72)	9315 (9.15)	15,420 (9.67)	
	Obese	69,758 (8.33)	6281 (5.3)	6708 (5.54)	7146 (6.41)	7783 (7.1)	10,878 (9.45)	10,875 (10.69)	20,087 (12.6)	
**Grade, n (%)**										
	7th	140,196 (16.75)	19,698 (16.63)	20,046 (16.56)	18,516 (16.62)	17,531 (15.99)	18,718 (16.25)	16,875 (16.58)	28,812 (18.07)	
	8th	145,377 (17.37)	20,953 (17.69)	21,030 (17.37)	19,272 (17.29)	18,982 (17.31)	18,990 (16.49)	17,324 (17.02)	28,826 (18.08)	
	9th	148,190 (17.71)	21,475 (18.13)	21,644 (17.88)	19,804 (17.77)	19,702 (17.97)	19,492 (16.92)	17,771 (17.46)	28,302 (17.75)	
	10th	132,805 (15.87)	18,780 (15.85)	19,190 (15.85)	17,531 (15.73)	17,055 (15.55)	19,018 (16.51)	15,835 (15.56)	25,396 (15.93)	
	11th	135,789 (16.22)	19,028 (16.06)	19,731 (16.3)	17,996 (16.15)	17,843 (16.27)	19,633 (17.05)	16,415 (16.13)	25,143 (15.77)	
	12th	134,615 (16.08)	18,523 (15.64)	19,438 (16.05)	18,313 (16.43)	18,541 (16.91)	19,318 (16.77)	17,551 (17.25)	22,931 (14.38)	
**Region of residence, n (%)**										
	Urban	541,928 (64.75)	71,150 (60.06)	75,778 (62.59)	74,454 (66.82)	72,549 (66.16)	76,221 (66.18)	67,554 (66.38)	104,222 (65.38)	
	Rural	295,044 (35.25)	47,307 (39.94)	45,301 (37.41)	36,978 (33.18)	37,105 (33.84)	38,948 (33.82)	34,217 (33.62)	55,188 (34.62)	
**Smoking status, n (%)**										
	No	798,876 (95.45)	102,201 (86.28)	111,177 (91.82)	108,595 (97.45)	105,948 (96.62)	113,177 (98.27)	99,929 (98.19)	157,849 (99.02)	
	Yes	38,096 (4.55)	16,256 (13.72)	9902 (8.18)	2837 (2.55)	3706 (3.38)	1992 (1.73)	1842 (1.81)	1561 (0.98)	
**Alcoholic consumption, n (%)**										
	No	693,100 (82.81)	90,006 (75.98)	94,937 (78.41)	91,157 (81.81)	91,744 (83.67)	97,895 (85)	86,087 (84.59)	141,274 (88.62)	
	Yes	143,872 (17.19)	28,451 (24.02)	26,142 (21.59)	20,275 (18.2)	17,910 (16.33)	17,274 (15)	15,684 (15.41)	18,136 (11.38)	
**School performance^c^, n (%)**										
	Low	95,889 (11.46)	15,897 (13.42)	15,551 (12.84)	14,929 (13.4)	12,301 (11.22)	11,507 (9.99)	10,056 (9.88)	15,648 (9.82)	
	Middle-low	199,758 (23.87)	30,782 (25.99)	31,193 (25.76)	28,149 (25.26)	25,943 (23.66)	25,751 (22.36)	22,255 (21.87)	35,685 (22.39)	
	Middle	235,371 (28.12)	31,917 (26.94)	32,365 (26.73)	29,912 (26.84)	30,137 (27.48)	32,783 (28.47)	29,891 (29.37)	48,366 (30.34)	
	Middle-high	203,302 (24.29)	26,665 (22.51)	28,639 (23.65)	26,227 (23.54)	27,243 (24.84)	29,369 (25.5)	25,768 (25.32)	39,391 (24.71)	
	High	102,652 (12.26)	13,196 (11.14)	13,331 (11.01)	12,215 (10.96)	14,030 (12.79)	15,759 (13.68)	13,801 (13.56)	20,320 (12.75)	
**Sexual experience, n (%)**										
	No	751,024 (89.73)	93,933 (79.3)	97,202 (80.28)	98,844 (88.7)	104,074 (94.91)	109,715 (95.26)	96,079 (94.41)	151,177 (94.84)	
	Yes	85,948 (10.27)	24,524 (20.7)	23,877 (19.72)	12,588 (11.3)	5580 (5.09)	5454 (4.74)	5692 (5.59)	8233 (5.16)	
**Physical activity frequency^d^, n (%)**										
	Lower activity	502,005 (59.98)	71,137 (60.05)	72,357 (59.76)	65,536 (58.81)	61,683 (56.25)	64,707 (56.18)	59,804 (58.76)	106,781 (66.99)	
	Moderate activity	207,881 (24.84)	31,427 (26.53)	32,204 (26.6)	30,804 (27.64)	30,540 (27.85)	31,667 (27.5)	25,261 (24.82)	25,978 (16.3)	
	Higher activity	127,086 (15.18)	15,893 (13.42)	16,518 (13.64)	15,092 (13.54)	17,431 (15.9)	18,795 (16.32)	16,706 (16.42)	26,651 (16.72)	
**Sadness and despair, n (%)**										
	No	585,417 (69.94)	72,615 (61.3)	77,976 (64.4)	76,928 (69.04)	81,804 (74.6)	86,103 (74.76)	73,455 (72.18)	116,536 (73.1)	
	Yes	251,555 (30.06)	45,842 (38.7)	43,103 (35.6)	34,504 (30.96)	27,850 (25.4)	29,066 (25.24)	28,316 (27.82)	42,874 (26.9)	
**Highest educational level of parents, n (%)**										
	High school or lower	424,689 (50.74)	49,612 (41.88)	56,851 (46.95)	55,905 (50.17)	60,585 (55.25)	67,515 (58.62)	52,916 (52)	81,305 (51)	
	College or higher	412,283 (49.26)	68,845 (58.12)	64,228 (53.05)	55,527 (49.83)	49,069 (44.75)	47,654 (41.38)	48,855 (48)	78,105 (49)	
**Family structure, n (%)**										
	Nuclear family	396,035 (47.32)	67,622 (57.09)	64,799 (53.52)	50,829 (45.61)	52,840 (48.19)	52,794 (45.84)	40,550 (39.84)	66,601 (41.78)	
	Living with relatives	14,831 (1.77)	3160 (2.67)	2862 (2.36)	2328 (2.09)	1843 (1.68)	1729 (1.50)	1269 (1.25)	1640 (1.03)	
	Living alone	6889 (0.82)	645 (0.54)	629 (0.52)	535 (0.48)	611 (0.56)	772 (0.67)	1322 (1.3)	2375 (1.49)	
	Orphanage	2786 (0.33)	398 (0.34)	431 (0.36)	372 (0.33)	417 (0.38)	379 (0.33)	327 (0.32)	462 (0.29)	

^a^KYRBS: Korea Youth Risk Behavior Web-Based Survey.

^b^BMI was divided into four groups according to the 2017 Korean National Growth Charts: underweight (<5%), normal (5% to 84%), overweight (85% to 94%), and obese (≥95%).

^c^School performance was divided into five groups: low (<20%), middle-low (20% to 39%), middle (40% to 59%), middle-high (60% to 79%), and high (≥80%).

^d^Physical activity frequency was divided into three groups based on the engagement in vigorous aerobic and resistance training more than three days per week: lower activity (neither activity is done for more than three days per week), moderate activity (either one activity), and higher activity (both activities).

**Figure 1:**
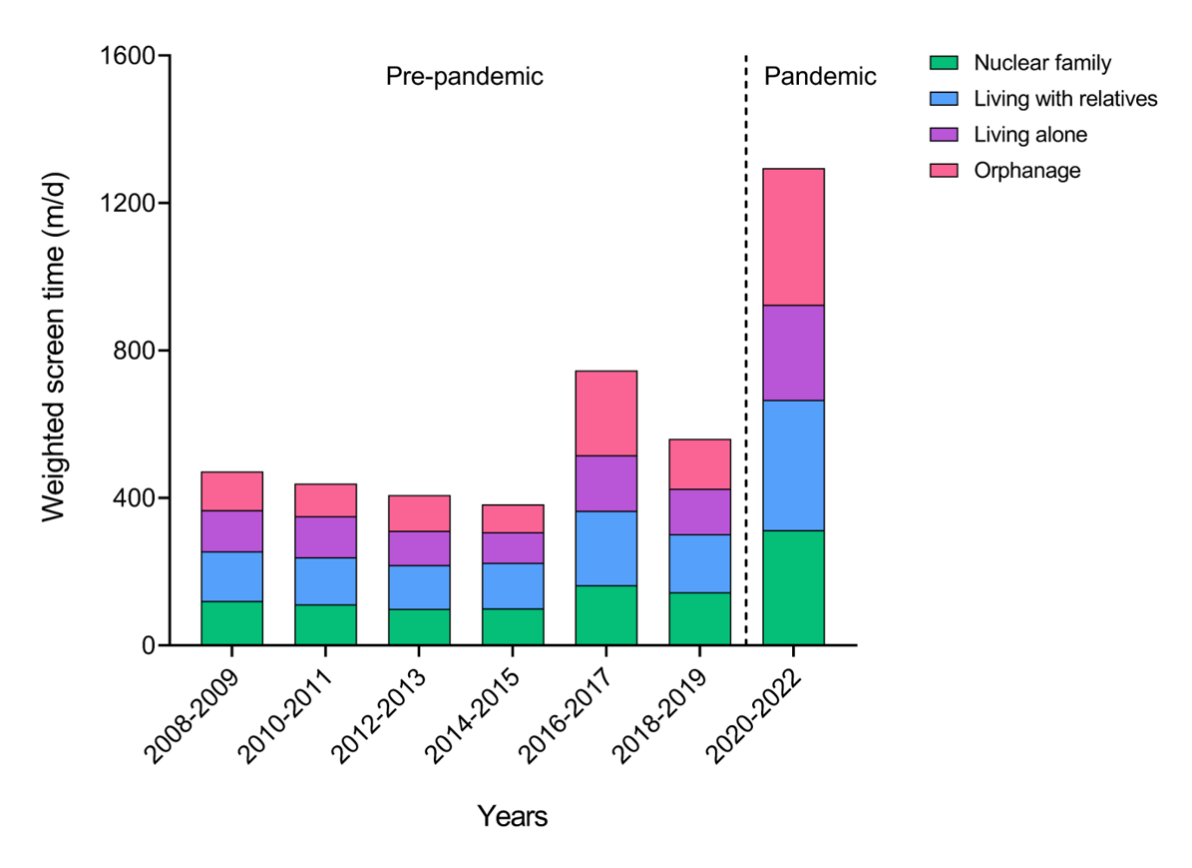
Trends in weighted screen time among adolescents in South Korea by family type, 2008-2022.

The average weekly screen time and the trend change before and during the pandemic for each demographic over 15 years are exhibited in Multimedia Appendix 1 and Tables S1-S4 in Multimedia Appendix 2. Particularly, the trend in average screen time for each family structure is illustrated in Figure 1. Screen time showed a consistent increase from 2008 to 2022. Starting at 119.80 minutes per day (m/d; 95% CI 118.63-120.98) in 2008-2009, it temporarily decreased to 99.64 m/d (95% CI 98.45-100.83) in 2012-2013, then increased to 165.68 m/d (95% CI 163.27-168.08) in 2016-2017, and finally reached 306.80 m/d (95% CI 304.30-309.30) in 2020-2022, reflecting a sharp increase during the pandemic.

The pandemic had a significant impact on screen time, as detailed in Tables S1-S4 in Multimedia Appendix 2. The analysis showed a modest increase in screen time during the prepandemic period (β=8.06, 95% CI 7.74-8.39) and a substantial rise during the pandemic (β=162.06, 95% CI 159.49-164.64), with a significant difference in growth rate (β_diff_=149.43, 95% CI 132.79-166.07). Among family structures, students in orphanages exhibited the largest increase (β_diff_=221.90, 95% CI 159.62-284.17).

**Figure 2:**
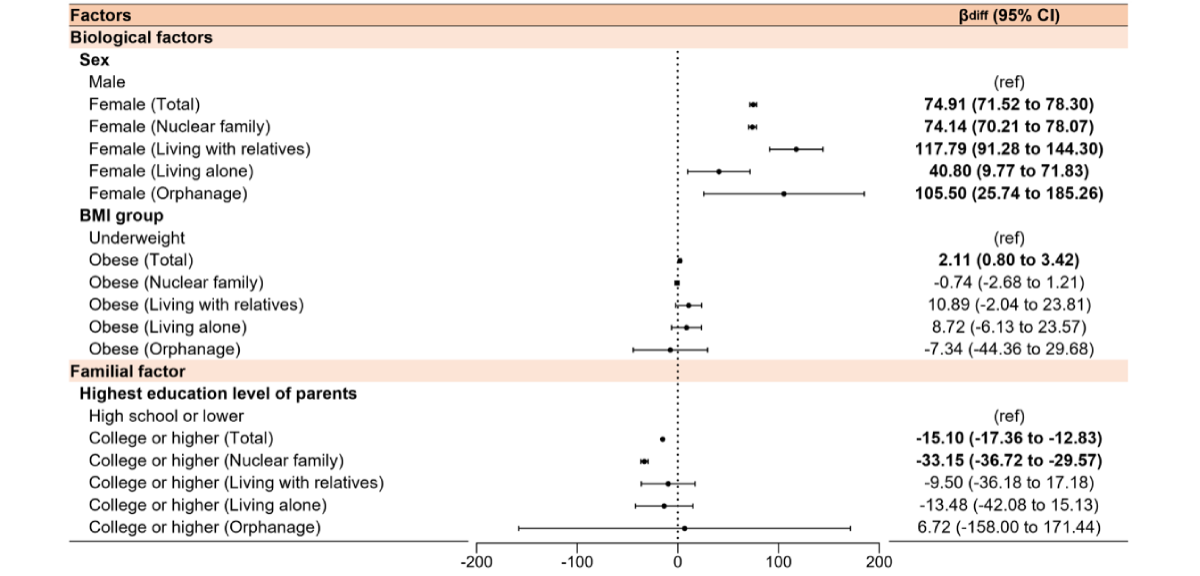
β differences of biological and familial factors in weighted average screen time among adolescents before and during the COVID-19 pandemic in KYRBS. KYRBS: Korea Youth Risk Behavior Web-Based Survey.

**Figure 3:**
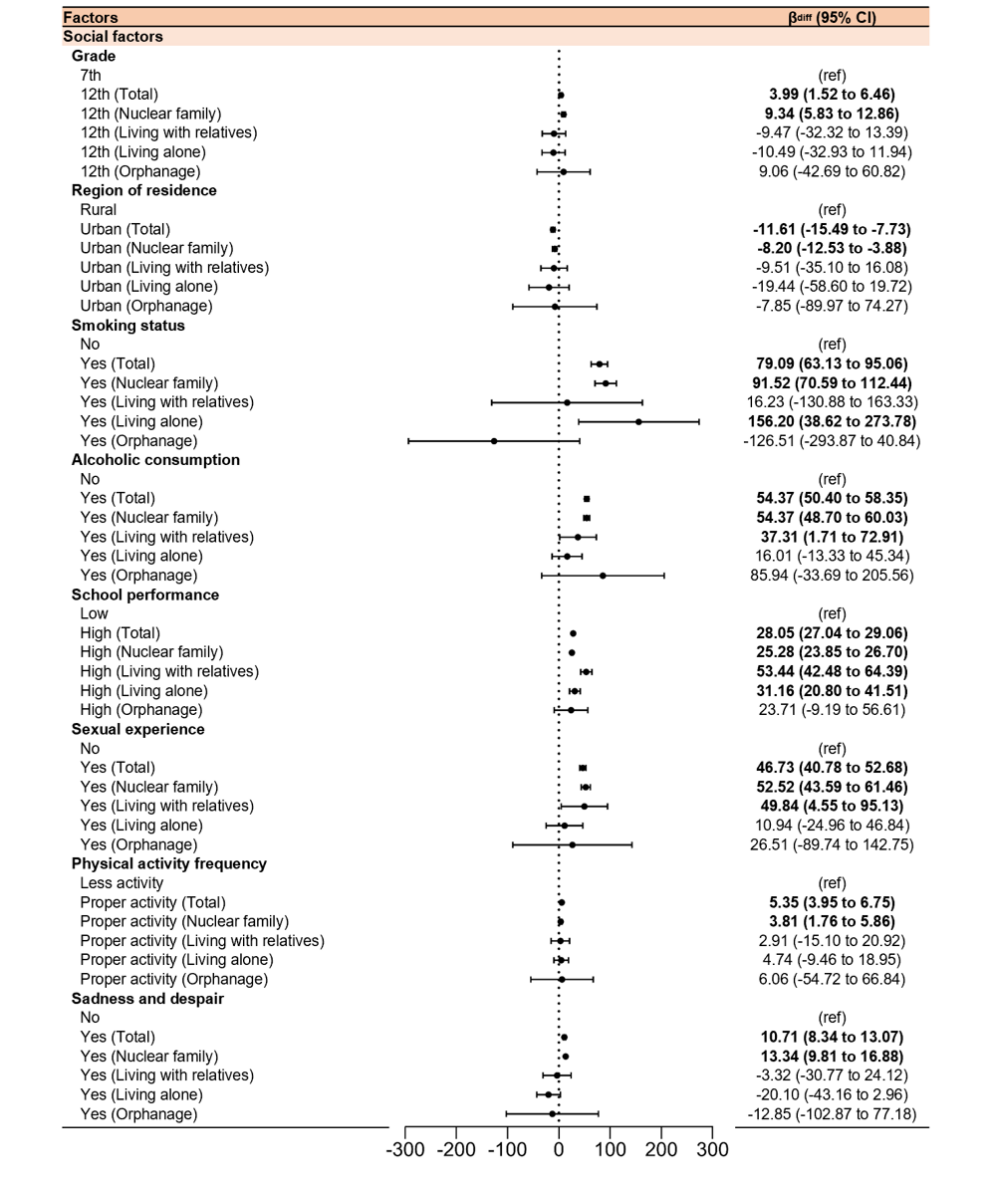
β differences of social factors in weighted average screen time among adolescents before and during the COVID-19 pandemic in KYRBS. KYRBS: Korea Youth Risk Behavior Web-Based Survey.

Table 2 highlights associations between screen time and biological, social, and familial factors. Women (β_diff_=74.91, 95% CI 71.52-78.30) and higher BMI (β_diff_=2.11, 95% CI 0.80-3.42) were identified as significant biological vulnerabilities. Among social factors, smoking (β_diff_=79.09, 95% CI 63.13-95.06), alcohol consumption (β_diff_=54.37, 95% CI 50.40-58.35), and sexual experience (β_diff_=46.73, 95% CI 40.78-52.68) had the strongest associations with increased screen time. Academic achievement (β_diff_=28.05, 95% CI 27.04-29.06]), rural residence (β_diff_=–11.61, 95% CI –15.49 to –7.73), sadness and despair (β_diff_ 10.71, 95% CI 8.34-13.07), higher physical activity (β_diff_=5.35, 95% CI 3.95-6.75), and higher grade (β_diff_=3.99, 95% CI 1.52-6.46) showed varying impacts (Figures 2 and 3).

**Table 2:** Factors associated with weighted average screen time among adolescents before and during the COVID-19 pandemic in KYRBS^a^.

Factors				Unadjusted model											Adjusted model^b^																
				Prepandemic screen time, β (95% CI)		Pandemic screen time, β (95% CI)		β_diff_ (95% CI)						Prepandemic screen time, β (95% CI)							Pandemic screen time, β (95% CI)						β_diff_ (95% CI)				
**Biological factors**																															
	**Sex**																														
		Men	1.00 (reference)		1.00 (reference)		1.00 (reference)						1.00 (reference)							1.00 (reference)						1.00 (reference)					
		Women	–5.51 (–6.82 to –4.19)^c^		61.18 (57.75 to 64.62)^c^		66.69 (63.01 to 70.38)^c^						–9.12 (–10.33 to –7.90)^c^							65.79 (62.63 to 68.95)^c^						74.91 (71.52 to 78.30)^c^					
	**BMI group^d^**																														
		Underweight	1.00 (reference)		1.00 (reference)		1.00 (reference)						1.00 (reference)							1.00 (reference)						1.00 (reference)					
		Obese	5.13 (4.65 to 5.61)^c^		2.61 (1.31 to 3.91)^c^		–2.52 (–3.90 to –1.13)^c^						3.51 (3.04 to 3.98)^c^							5.62 (4.40 to 6.85)^c^						2.11 (0.80 to 3.42)^c^					
**Social factors**																															
	**Grade**																														
		7th	1.00 (reference)		1.00 (reference)		1.00 (reference)						1.00 (reference)							1.00 (reference)						1.00 (reference)					
		12th	–0.09 (–0.40 to 0.22)		9.43 (8.18 to 10.68)^c^		9.52 (8.24 to 10.81)^c^						–5.65 (–6.31 to –4.98)^c^							–1.66 (–4.04 to 0.72)						3.99 (1.52 to 6.46)^c^					
	**Region of residence**																														
		Rural	1.00 (reference)		1.00 (reference)					1.00 (reference)							1.00 (reference)						1.00 (reference)						1.00 (reference)		
		Urban	–0.79 (–2.09 to 0.51)		–16.20 (–21.03 to –11.38)^c^					–15.41 (–20.41 to –10.41)^c^							1.08 (–0.13 to 2.29)						–10.53 (–14.22 to –6.84)^c^						–11.61 (–15.49 to –7.73)^c^		
	**Smoking status**																														
		No	1.00 (reference)		1.00 (reference)					1.00 (reference)							1.00 (reference)						1.00 (reference)						1.00 (reference)		
		Yes	9.10 (7.27 to 10.93)^c^		174.49 (158.20 to 190.79)^c^					165.39 (148.99 to 181.79)^c^							6.46 (4.55 to 8.38)^c^						85.55 (69.70 to 101.41)^c^						79.09 (63.13 to 95.06)^c^		
	**Alcoholic consumption**																														
		No	1.00 (reference)		1.00 (reference)						1.00 (reference)							1.00 (reference)						1.00 (reference)						1.00 (reference)	
		Yes	14.79 (13.75 to 15.83)^c^		95.80 (91.51 to 100.08)^c^						81.01 (76.60 to 85.42)^c^							13.89 (12.85 to 14.93)^c^						68.26 (64.42 to 72.10)^c^						54.37 (50.40 to 58.35)^c^	
	**School performance^e^**																														
		Low	1.00 (reference)		1.00 (reference)							1.00 (reference)							1.00 (reference)						1.00 (reference)						1.00 (reference)
		High	10.15 (9.84 to 10.46)^c^		43.33 (42.33 to 44.33)^c^							33.18 (32.14 to 34.23)^c^							9.29 (8.97 to 9.60)^c^						37.34 (36.38 to 38.30)^c^						28.05 (27.04 to 29.06)^c^
	**Sexual experience**																														
		No	1.00 (reference)		1.00 (reference)					1.00 (reference)							1.00 (reference)						1.00 (reference)						1.00 (reference)		
		Yes	–0.31 (–1.55 to 0.94)		88.53 (82.21 to 94.86)^c^					88.84 (82.40 to 95.28)^c^							0.92 (–0.30 to 2.14)						47.65 (41.83 to 53.47)^c^						46.73 (40.78 to 52.68)^c^		
	**Physical activity frequency^f^**																														
		Lower activity	1.00 (reference)		1.00 (reference)				1.00 (reference)							1.00 (reference)						1.00 (reference)						1.00 (reference)			
		Higher activity	–3.96 (–4.47 to –3.44)^c^		–11.69 (–13.17 to –10.22)^c^		–7.74 (–9.30 to –6.17)^c^						–7.37 (–7.84 to –6.91)^c^							–2.02 (–3.34 to –0.71)^c^						5.35 (3.95 to 6.75)^c^					
	**Sadness and despair**																														
		No	1.00 (reference)		1.00 (reference)		1.00 (reference)						1.00 (reference)							1.00 (reference)						1.00 (reference)					
		Yes	6.91 (6.17 to 7.66)^c^		44.01 (41.61 to 46.42)^c^		37.10 (34.58 to 39.62)^c^						7.84 (7.11 to 8.57)^c^							18.55 (16.29 to 20.80)^c^						10.71 (8.34 to 13.07)^c^					
**Familial factor**																															
	**Highest education level of parents**																														
		High school or lower	1.00 (reference)		1.00 (reference)		1.00 (reference)						1.00 (reference)							1.00 (reference)						1.00 (reference)					
		College or higher	–14.11 (–14.88 to –13.34)^c^		–41.55 (–43.79 to –39.30)^c^		–27.43 (–29.81 to –25.06)^c^						–11.22 (–11.97 to –10.46)^c^							–26.31 (–28.45 to –24.17)^c^						–15.10 (–17.36 to –12.83)^c^					

^a^KYRBS: Korea Youth Risk Behavior Web-Based Survey.

^b^Adjustment for age, sex, BMI group, grade, region of residence, smoking status, alcoholic consumption, school performance, sexual experience, physical activity frequency, sadness and despair, and highest education level of parents.

^c^Indicate a significant difference (*P*<.05).

^d^BMI was divided into four groups according to the 2017 Korean National Growth Charts: underweight (<5%), normal (5% to 84%), overweight (85% to 94%), and obese (≥95%). BMI was calculated as weight in kilograms divided by height in meters squared.

^e^School performance was divided into five groups: low (<20%), middle-low (20% to 39%), middle (40% to 59%), middle-high (60% to 79%), and high (≥80%).

^f^Physical activity frequency was divided into three groups based on the engagement in vigorous aerobic and resistance training more than three days per week: lower activity (neither activity is done for more than three days per week), moderate activity (either one activity), and higher activity (both activities).

Adjusted models presented in Tables S5-S8 in Multimedia Appendix 2 explored associations across family structures. Before the pandemic, men in nuclear families (β=–10.50, 95% CI –11.92 to –9.08) and those living with relatives (β=–15.14, 95% CI –21.58 to –8.71) had lower screen time. In contrast, women living alone (β=14.80, 95% CI 3.57-26.03) showed higher screen time. During the pandemic, screen time significantly increased for women across all family structures: nuclear families (β_diff_=74.14, 95% CI 70.21-78.07), living with relatives (β_diff_ 117.79, 95% CI 91.28-144.30), living alone (β_diff_ 40.80, 95% CI 9.77-71.83), and in orphanages (β_diff_ 105.50, 95% CI 25.74-185.26; Figure 3).

## Discussion

### Key Findings

**Figure 4:**
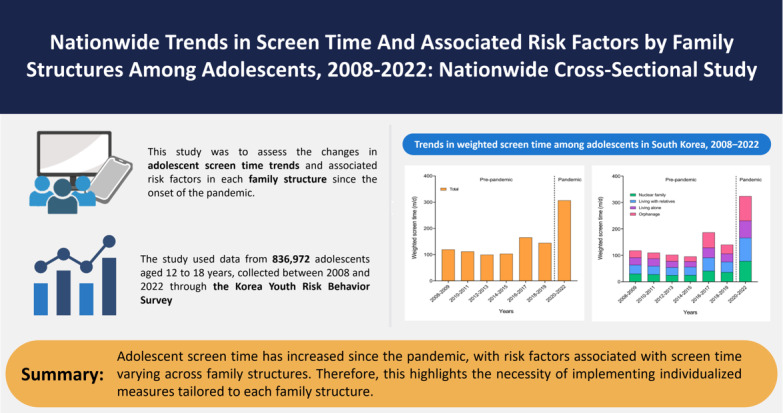
Graphical conclusion.

This study stands out as the initial comprehensive long-term trend analysis of adolescent screen time and associated factors across various family structures, encompassing the COVID-19 era (Figure 4). There was an overall increase in screen time from 2008 to 2022. Specifically, throughout the pandemic spanning from 2020 to 2022, there was a substantial increase in adolescent screen time compared to the prepandemic. Among various family structures, students within the orphanage group exhibited the most significant increase in screen time since the pandemic onset. All examined factors exhibited statistically significant alterations in their association with adolescent screen time during the pandemic. Particularly, stratified by family structure, it was consistently observed that women had more vulnerability to increased screen time during the pandemic regardless of family structure. Meanwhile, the nuclear family group showed distinctive screen time-associated factors, including grade, region of residence, physical activity frequency, sadness and despair, and highest education level of parents, which were not present in the other family groups.

### Comparison With Previous Studies

Earlier investigations have predominantly focused on the changes in adolescent screen time trends during the COVID-19 pandemic. Studies conducted in countries such as Canada (n=29,027), Germany (n=1711), the United States (n=5412), China (n=1680), and South Korea (n=913,191) consistently reported significant increases in screen time following the onset of the pandemic [14,26-28]. Many of these studies also examined factors influencing screen time, including depression and mental health [10,11]. However, most were limited by relatively small sample sizes, restricted age ranges of participants, or insufficient analysis of trends across specific demographic subgroups, such as those defined by family structure [26-29].

Additionally, while existing studies from Norway (n=4509) and France (n=3720) have explored the association between adolescent screen time and family structure, they often lack diversity in the types of family structures considered and fail to account for the profound shifts introduced by the pandemic [15,16]. These limitations highlight the importance of considering a wider range of family structures and the influence of contextual factors like the pandemic when analyzing screen time trends [27,28].

In contrast, this study addresses these gaps by analyzing a significantly larger sample size (n=836,972) collected over 15 years, allowing for a more robust examination of long-term trends. Furthermore, the stratification of adolescents by diverse family structures—nuclear families, living with relatives, living alone, and orphanages—enables a unique comparative analysis of disparities in screen time trends across these groups. This approach not only provides a more comprehensive understanding of the changes in adolescent screen time but also sheds light on the distinct risk factors associated with different family structures during the pandemic.

### Plausible Mechanism

The overall elevation in average screen time of adolescents spanning from 2008 to 2022 could be elucidated by the rise in smartphone penetration among adolescents. The prevalence of smartphones among adolescents in South Korea increased by 15.3%, rising from 79.3% in 2015 to 94.6% in 2022 [30]. This upward trend would have played a role in the elevation of screen time among Korean adolescents over the 15 years. Particularly, a substantial increase in screen time with the onset of the pandemic is likely due to the increased duration of indoor activities. During the pandemic, there was an increase in time spent indoors at home and a decrease in outdoor activities, including walking, cycling, and commuting [31]. Students were restricted from the absence of the school community covering a range of cultural, sports, social, and emotional experiences. Instead, there was an increase in internet use time for video watching and gaming with the onset of the pandemic, suggesting an influence on the rapid rise in screen time [32]. Fundamentally, the rise in screen time could be attributed to the shift in the pandemic lifestyles of students: decreased outdoor activities and various web-based engagements.

Across all family structures, a marked increase in screen time was evident following the pandemic. However, there was a specific increase in screen time among students belonging to the orphanage group. This suggests a potential influence of the presence of parents or relatives on screen time during the pandemic. The Royal College of Paediatrics and Child Health emphasized the significance of the roles of guardians in managing adolescent screen time. They recommended careful parental consideration when introducing technology to adolescents and setting individualized regulations for their screen time [16]. This detailed assistance, initially inadequate for the orphanage group, became further deficient during the pandemic due to the obstruction of support from teachers in schools. It is likely to have contributed to the substantial screen time adjustment failure observed in the orphanage group.

We analyzed the association between various biological, social, and familial factors and screen time according to the family structure. Among the diverse family structures, the nuclear family group exhibited several unique screen time-associated factors from the onset of the pandemic that were not observed in other family structures. In particular, the region of residence indicated that students residing in urban regions were less susceptible to increased screen time during the pandemic compared to their counterparts in rural regions. This finding is likely linked to parental abilities associated with the region of residence. Parents have been identified as having heightened concerns related to screen-related risks, such as cyberbullying and exposure to inappropriate or harmful content [33]. The perceived difference in screen time between regions is likely because parents residing in urban regions exhibited higher levels of parenting compared to those living in rural regions [34]. Particularly, the increase in indoor time during the pandemic induced an increase in the time spent with parents, indicating an intensified influence of parenting on adolescents. Therefore, it may be that students residing in urban regions were less vulnerable to increased screen time during the pandemic than students residing in rural regions.

### Clinical and Policy Implications

This study offers a reminder of the recent rapid increase in screen time among adolescents. Considering the rise in screen time since the pandemic, it is imperative for clinical professionals and policymakers to promptly propose relevant regulation solutions. As a noticeable increase in screen time has been observed among adolescents belonging to the orphanage group following the pandemic, officials should implement more intensive management strategies for this particular group. Heightened management is also required for women adolescents, who exhibited vulnerability to increased screen time across all family structures during the pandemic. We additionally suggest the development of a customized screen time management strategy tailored to the specific family structures of each adolescent. Providing individualized solutions targeting the risk factors associated with each family structure can contribute to a more effective reduction in screen time. Specifically, focusing on the distinct risk factors (grade, region of residence, physical activity frequency, sadness and despair, and highest education level of parents) within the nuclear family group could prompt targeted management strategies for this particular cohort. Given the physical and mental health and socioemotional well-being concerns associated with excessive screen time [10,35,36], appropriate efforts from clinical, social, and political professionals are needed to normalize the rapidly increased screen time among adolescents since the pandemic. To address this, it is essential to expand international health expenditure for pandemic response and preparedness [37]. Thus, securing long-term and sustainable funding is crucial to address the rise in adolescents’ digital device use and the associated mental health challenges [38].

In particular, investments in digital education programs, enhanced mental health services, and community support networks are vital to mitigate the physical and mental health impacts of increased screen time among adolescents during the pandemic. Additionally, policy and educational initiatives should focus on leveraging the already identified positive aspects of increased screen time during restricted social interactions, such as its potential for information exchange, education, and communication [39]. Pandemic response funds should be prioritized to address these issues and support adolescents in growing up in a healthy digital environment. Appropriate allocation of funds and policy support will play a critical role in addressing the challenges of increased adolescent screen time and alleviating health disparities exacerbated by the pandemic.

### Limitations

This study has several limitations. First, variations in survey questions from 2008 to 2022 affected data comparability. Specifically, while smartphone use time was the focus in 2017 and from 2020 to 2022, other years emphasized internet use time, excluding total screen time such as television viewing. Nevertheless, to the best of our knowledge, this is the first study to analyze long-term data spanning 15 years, providing comprehensive insights into prepandemic and pandemic-era trends in adolescent screen time. Second, the self-response methodology in KYRBS may not fully reflect the characteristics of the actual population. However, KYRBS has been historically acknowledged as a credible dataset [40,41], and the weight-based analysis conducted with over 800,000 survey respondents further enhances the representativeness and validity of the screen time trends observed in this study. Third, this study is limited to Korean adolescents, restricting its representation of global trends. Despite this, the focus on a specific demographic allowed us to examine the nuanced relationships between screen time and family structures in the Korean context, providing insights relevant to similar societies. Finally, the cross-sectional design prevents establishing causal relationships between screen time and associated factors, highlighting the need for longitudinal and comprehensive studies. However, the study emphasizes the need for longitudinal research and offers a foundational understanding of screen time trends and influencing factors across diverse family structures.

### Conclusions

This study examined adolescent screen time trends from 2008 to 2022 across various family structures, revealing a consistent increase over time, with the steepest rise observed during the COVID-19 pandemic. Screen time patterns varied significantly by family structure, with orphaned adolescents experiencing the most pronounced increase, while other groups, such as those in nuclear families, also showed distinct trends influenced by factors like parental education and region of residence. These findings underscore the importance of tailored interventions, including family-type–specific workshops, institutional programs, and digital literacy resources, to address the diverse needs of adolescents. Reducing regional disparities and promoting alternative activities are also critical for mitigating the negative impacts of excessive screen time. Future research on broader structural and cultural influences on digital engagement is necessary to develop comprehensive and sustainable strategies for managing adolescent screen time.

## Appendix

Multimedia Appendix 1 The trend in the average screen time (m/d) of adolescents before and during the COVID-19 pandemic, weighted mean (95% CI), in the Korea Youth Risk Behavior Web-Based Survey.

Multimedia Appendix 2 Trends and factors associated with adolescent screen time before and during the COVID-19 pandemic by household type in the Korea Youth Risk Behavior Web-Based Survey.

## Abbreviations

KYRBS: Korea Youth Risk Behavior Web-Based Survey

## Data Availability

The datasets generated or analyzed during this study are available from the Korea Disease Control and Prevention Agency repository [42].
