# Supplementary material for: Nationwide Trends in Screen Time and Associated Risk Factors by Family Structures Among Adolescents, 2008-2022: Nationwide Cross-Sectional Study
Source: JMIR Public Health Surveill. 2025 Mar 10;11:e57962. doi: 10.2196/57962 (PMC11933748; doi:10.2196/57962)
Supplement: Multimedia Appendix 1 [file publichealth_v11i1e57962_app1.docx]

|  | Pre-pandemic | | | | | | Pandemic | Trends in the pre-pandemic, β (95% CI) | Trends in the pandemic, β　(95% CI) | Trend differences, β_diff_ (95% CI) |
| --- | --- | --- | --- | --- | --- | --- | --- | --- | --- | --- |
| Year | 2008-2009 | 2010-2011 | 2012-2013 | 2014-2015 | 2016-2017 | 2018-2019 | 2020-2022 |  |  |  |
| Overall | 119.80 (118.63 to 120.98) | 112.02 (110.97 to 113.07) | 99.64 (98.45 to 100.83) | 103.52 (102.32 to 104.71) | 165.68 (163.27 to 168.08) | 144.73 (143.46 to 146.01) | 306.80 (304.30 to 309.30) | **8.06 (7.74 to 8.39)** | **162.06 (159.49 to 164.64)** | **149.43 (132.79 to 166.07)** |
| Sex | | | | | | | | | | |
| Men | 124.63 (123.04 to 126.22) | 115.12 (113.62 to 116.62) | 115.25 (113.71 to 116.79) | 117.37 (115.77 to 118.97) | 152.33 (149.73 to 154.92) | 135.69 (134.17 to 137.21) | 277.13 (274.38 to 279.87) | **4.77 (4.36 to 5.18)** | **141.44 (138.49 to 144.38)** | **130.98 (120.40 to 141.55)** |
| Women | 114.12 (112.71 to 115.54) | 108.40 (107.21 to 109.58) | 81.26 (80.14 to 82.39) | 88.16 (86.87 to 89.46) | 180.10 (176.23 to 183.98) | 154.23 (152.57 to 155.88) | 338.31 (335.28 to 341.34) | **11.81 (11.31 to 12.31)** | **184.09 (180.85 to 187.33)** | **164.418 (140.075 to 188.761)** |
| BMI group ^a^ | | | | | | | | | | |
| Underweight | 122.44 (119.78 to 125.10) | 114.34 (111.84 to 116.85) | 103.52 (100.63 to 106.41) | 109.34 (106.32 to 112.36) | 164.34 (159.66 to 169.01) | 143.05 (139.83 to 146.27) | 304.08 (299.69 to 308.47) | **6.69 (5.98 to 7.41)** | **161.03 (156.03 to 166.04)** | **157.09 (141.74 to 172.45)** |
| Normal | 117.48 (116.28 to 118.68) | 109.88 (108.79 to 110.97) | 95.75 (94.57 to 96.93) | 99.81 (98.58 to 101.04) | 162.28 (159.82 to 164.74) | 142.61 (141.24 to 143.98) | 302.10 (299.56 to 304.65) | **7.84 (7.50 to 8.17)** | **159.49 (156.84 to 162.15)** | **145.91 (129.67 to 162.15)** |
| Overweight | 123.80 (120.93 to 126.67) | 120.21 (117.20 to 123.22) | 107.59 (104.90 to 110.28) | 110.24 (107.52 to 112.96) | 171.34 (167.03 to 175.65) | 148.91 (145.85 to 151.96) | 303.50 (299.37 to 307.63) | **8.38 (7.67 to 9.09)** | **154.59 (149.88 to 159.30)** | **129.40 (110.98 to 147.82)** |
| Obese | 135.44 (131.65 to 139.23) | 127.27 (124.06 to 130.47) | 124.27 (121.10 to 127.45) | 125.33 (122.12 to 128.55) | 178.23 (173.97 to 182.48) | 155.58 (152.60 to 158.56) | 325.93 (321.97 to 329.88) | **7.97 (7.18 to 8.75)** | **170.35 (165.81 to 174.89)** | **148.15 (130.41 to 165.90)** |
| Grade | | | | | | | | | | |
| 7^th^ | 110.62 (108.56 to 112.68) | 103.68 (101.73 to 105.63) | 85.24 (83.26 to 87.22) | 95.83 (93.58 to 98.09) | 152.77 (149.50 to 156.04) | 148.45 (145.82 to 151.07) | 268.79 (265.04 to 272.55) | **9.30 (8.74 to 9.85)** | **120.34 (116.09 to 124.60)** | **114.34 (94.56 to 134.12)** |
| 8^th^ | 121.73 (119.61 to 123.84) | 115.18 (112.92 to 117.44) | 102.25 (100.13 to 104.37) | 109.60 (107.21 to 112.00) | 175.43 (171.47 to 179.38) | 157.95 (155.23 to 160.66) | 298.84 (295.15 to 302.53) | **9.87 (9.27 to 10.47)** | **140.89 (136.63 to 145.14)** | **129.14 (108.19 to 150.10)** |
| 9^th^ | 128.86 (126.57 to 131.15) | 120.73 (118.76 to 122.70) | 110.70 (108.32 to 113.08) | 110.95 (108.66 to 113.23) | 175.10 (171.31 to 178.88) | 156.46 (153.76 to 159.17) | 319.48 (315.69 to 323.26) | **8.00 (7.40 to 8.60)** | **163.01 (158.69 to 167.34)** | **149.38 (131.97 to 166.78)** |
| 10^th^ | 117.66 (115.34 to 119.97) | 112.65 (110.39 to 114.90) | 96.87 (94.47 to 99.27) | 99.75 (97.40 to 102.10) | 158.46 (154.29 to 162.63) | 142.38 (139.66 to 145.10) | 310.71 (306.23 to 315.20) | **7.43 (6.81 to 8.05)** | **168.33 (163.56 to 173.10)** | **150.11 (133.63 to 166.59)** |
| 11^th^ | 118.26 (115.81 to 120.71) | 110.64 (108.41 to 112.88) | 104.07 (101.62 to 106.51) | 107.33 (104.71 to 109.96) | 173.64 (168.93 to 178.34) | 136.62 (133.90 to 139.34) | 322.37 (317.68 to 327.06) | **8.50 (7.83 to 9.16)** | **185.75 (180.82 to 190.67)** | **171.26 (152.64 to 189.89)** |
| 12^th^ | 120.61 (117.93 to 123.29) | 108.16 (105.72 to 110.59) | 97.34 (94.96 to 99.73) | 96.47 (94.06 to 98.88) | 158.94 (154.11 to 163.77) | 130.23 (127.65 to 132.80) | 322.15 (316.98 to 327.32) | **5.91 (5.24 to 6.57)** | **191.92 (186.67 to 197.18)** | **176.24 (162.33 to 190.15)** |
| Region of residence | | | | | | | | | | |
| Urban | 119.75 (118.27 to 121.22) | 111.64 (110.32 to 112.96) | 100.33 (98.93 to 101.73) | 104.36 (102.89 to 105.83) | 163.35 (160.67 to 166.03) | 144.83 (143.29 to 146.37) | 301.81 (298.75 to 304.87) | **7.88 (7.50 to 8.27)** | **156.98 (153.84 to 160.11)** | **144.12 (127.62 to 160.62)** |
| Rural | 119.94 (118.08 to 121.80) | 112.87 (111.21 to 114.54) | 98.05 (95.77 to 100.33) | 101.59 (99.54 to 103.64) | 170.93 (165.99 to 175.87) | 144.51 (142.24 to 146.78) | 318.01 (313.77 to 322.25) | **8.47 (7.86 to 9.08)** | **173.50 (169.05 to 177.94)** | **157.28 (138.93 to 175.63)** |
| Smoking status | | | | | | | | | | |
| No | 117.06 (115.91 to 118.21) | 111.59 (110.51 to 112.68) | 99.24 (98.05 to 100.43) | 103.10 (101.90 to 104.30) | 164.62 (162.22 to 167.01) | 144.84 (143.56 to 146.12) | 305.04 (302.55 to 307.54) | **8.71 (8.39 to 9.04)** | **160.20 (157.63 to 162.78)** | **144.63 (126.73 to 162.53)** |
| Yes | 137.19 (134.41 to 139.98) | 116.64 (113.45 to 119.83) | 114.18 (108.33 to 120.03) | 114.50 (109.27 to 119.72) | 221.90 (207.42 to 236.38) | 139.55 (131.20 to 147.90) | 479.53 (462.50 to 496.56) | **4.03 (2.58 to 5.48)** | **339.99 (321.24 to 358.73)** | **324.71 (303.10 to 346.31)** |
| Alcoholic consumption | | | | | | | | | | |
| No | 115.85 (114.67 to 117.03) | 109.58 (108.45 to 110.71) | 97.64 (96.43 to 98.86) | 101.92 (100.71 to 103.14) | 157.83 (155.57 to 160.09) | 143.89 (142.60 to 145.18) | 295.81 (293.42 to 298.20) | **8.23 (7.91 to 8.55)** | **151.91 (149.42 to 154.41)** | **137.90 (120.95 to 154.84)** |
| Yes | 132.52 (130.44 to 134.59) | 120.89 (119.02 to 122.76) | 108.63 (106.50 to 110.75) | 111.34 (108.92 to 113.77) | 208.14 (202.89 to 213.39) | 149.15 (146.38 to 151.93) | 391.60 (386.44 to 396.77) | **8.90 (8.24 to 9.57)** | **242.45 (237.08 to 247.82)** | **227.48 (207.98 to 246.99)** |
| School performance ^b^ | | | | | | | | | | |
| Low | 142.05 (139.26 to 144.83) | 131.87 (129.34 to 134.39) | 115.07 (112.51 to 117.62) | 122.30 (119.38 to 125.23) | 215.54 (209.59 to 221.49) | 164.11 (160.06 to 168.16) | 411.81 (406.78 to 416.84) | **9.82 (8.97 to 10.67)** | **247.70 (241.78 to 253.62)** | **228.51 (206.77 to 250.25)** |
| Middle-low | 129.51 (127.78 to 131.23) | 121.78 (120.28 to 123.27) | 108.12 (106.35 to 109.88) | 112.72 (110.82 to 114.62) | 187.32 (183.81 to 190.84) | 156.23 (153.94 to 158.51) | 353.08 (349.85 to 356.31) | **9.04 (8.53 to 9.54)** | **196.85 (193.22 to 200.48)** | **184.32 (165.26 to 203.37)** |
| Middle | 115.70 (114.16 to 117.24) | 108.15 (106.70 to 109.60) | 96.94 (95.36 to 98.52) | 101.31 (99.67 to 102.94) | 162.84 (160.07 to 165.60) | 144.64 (142.89 to 146.38) | 301.43 (298.75 to 304.10) | **8.87 (8.46 to 9.27)** | **156.79 (153.86 to 159.72)** | **143.41 (125.00 to 161.83)** |
| Middle-high | 111.18 (109.63 to 112.74) | 104.75 (103.22 to 106.27) | 92.99 (91.43 to 94.55) | 97.59 (96.00 to 99.19) | 148.67 (146.09 to 151.26) | 138.80 (136.97 to 140.63) | 267.57 (264.92 to 270.22) | **7.78 (7.37 to 8.18)** | **128.76 (125.80 to 131.72)** | **118.11 (101.80 to 134.42)** |
| High | 98.63 (96.19 to 101.07) | 91.49 (89.36 to 93.63) | 82.68 (80.48 to 84.89) | 86.31 (84.24 to 88.39) | 130.38 (127.16 to 133.60) | 122.99 (120.53 to 125.44) | 234.02 (230.64 to 237.40) | **6.96 (6.40 to 7.52)** | **111.03 (107.19 to 114.88)** | **103.82 (88.58 to 119.05)** |
| Sexual experience | | | | | | | | | | |
| No | 118.28 (117.09 to 119.48) | 111.21 (110.15 to 112.27) | 99.10 (97.91 to 100.29) | 103.66 (102.45 to 104.86) | 163.52 (161.14 to 165.91) | 145.51 (144.24 to 146.79) | 302.09 (299.65 to 304.54) | **8.66 (8.33 to 8.99)** | **156.58 (154.04 to 159.11)** | **143.53 (125.73 to 161.32)** |
| Yes | 125.62 (123.48 to 127.77) | 115.25 (113.22 to 117.28) | 103.81 (100.94 to 106.68) | 101.00 (96.89 to 105.11) | 206.52 (197.44 to 215.60) | 132.20 (127.60 to 136.79) | 390.62 (383.21 to 398.04) | **5.61 (4.65 to 6.57)** | **258.43 (250.45 to 266.41)** | **241.58 (226.42 to 256.74)** |
| Physical activity frequency ^c^ | | | | | | | | | | |
| Lower activity | 121.79 (120.52 to 123.07) | 114.26 (113.06 to 115.46) | 99.77 (98.36 to 101.19) | 103.35 (101.95 to 104.75) | 170.97 (167.93 to 174.00) | 150.81 (149.33 to 152.28) | 313.33 (310.62 to 316.04) | **8.73 (8.35 to 9.11)** | **162.53 (159.69 to 165.36)** | **147.06 (128.98 to 165.14)** |
| Moderate activity | 117.88 (116.12 to 119.63) | 110.60 (108.99 to 112.21) | 99.79 (98.23 to 101.35) | 105.90 (104.19 to 107.60) | 158.23 (155.51 to 160.94) | 139.51 (137.57 to 141.44) | 293.31 (289.81 to 296.82) | **7.24 (6.79 to 7.68)** | **153.80 (150.13 to 157.48)** | **142.14 (126.65 to 157.63)** |
| Higher activity | 114.64 (112.39 to 116.89) | 105.02 (102.98 to 107.06) | 98.75 (96.61 to 100.89) | 99.93 (97.67 to 102.19) | 159.46 (155.99 to 162.94) | 130.28 (127.94 to 132.61) | 292.71 (289.33 to 296.09) | **7.15 (6.60 to 7.71)** | **162.43 (158.66 to 166.20)** | **149.54 (133.97 to 165.11)** |
| Sadness and despair | | | | | | | | | | |
| No | 117.07 (115.78 to 118.36) | 109.98 (108.80 to 111.15) | 99.84 (98.55 to 101.14) | 102.80 (101.48 to 104.12) | 158.60 (156.25 to 160.95) | 140.36 (139.02 to 141.70) | 294.97 (292.56 to 297.39) | **7.77 (7.43 to 8.10)** | **154.61 (152.07 to 157.15)** | **139.52 (123.43 to 155.61)** |
| Yes | 124.14 (122.52 to 125.75) | 115.74 (114.30 to 117.17) | 99.18 (97.58 to 100.79) | 105.61 (103.84 to 107.38) | 186.48 (182.92 to 190.04) | 156.00 (153.85 to 158.15) | 338.99 (335.55 to 342.42) | **9.51 (9.03 to 9.99)** | **182.98 (179.27 to 186.70)** | **168.83 (148.89 to 188.77)** |
| Highest educational level of parents | | | | | | | | | | |
| High school or lower | 108.20 (106.86 to 109.54) | 103.21 (102.01 to 104.40) | 93.40 (92.14 to 94.65) | 98.70 (97.43 to 99.96) | 153.61 (151.17 to 156.05) | 143.39 (141.90 to 144.89) | 287.05 (284.54 to 289.57) | **9.80 (9.44 to 10.16)** | **143.66 (140.98 to 146.35)** | **127.51 (107.42 to 147.60)** |
| College or higher | 129.40 (128.02 to 130.79) | 120.76 (119.44 to 122.07) | 106.50 (104.95 to 108.05) | 109.86 (108.22 to 111.50) | 183.97 (180.77 to 187.17) | 146.26 (144.55 to 147.98) | 328.60 (325.71 to 331.49) | **7.01 (6.59 to 7.43)** | **182.34 (179.25 to 185.42)** | **168.33 (153.45 to 183.21)** |

Abbreviations: BMI, body mass index (calculated as weight in kilograms divided by height in meters squared); CI, confidence interval; KYRBS, Korea Youth Risk Behavior Web-Based Survey.

Numbers in bold indicate a significant difference (*P* < 0.05).

^a^ BMI was divided into four groups according to the 2017 Korean National Growth Charts: underweight (<5%), normal (5% to 84%), overweight (85% to 94%), and obese (≥95%).

^b^ School performance was divided into five groups: low (<20%), middle-low (20% to 39%), middle (40% to 59%), middle-high (60% to 79%), and high (≥80%).

^c^ Physical activity frequency was divided into the three groups based on the engagement in vigorous aerobic and resistance trainings more than three days per week: lower activity (neither activity is done for more than three days per week), moderate activity (either one activity), and higher activity (both activities).
