# Supplementary material for: Nationwide Trends in Screen Time and Associated Risk Factors by Family Structures Among Adolescents, 2008-2022: Nationwide Cross-Sectional Study
Source: JMIR Public Health Surveill. 2025 Mar 10;11:e57962. doi: 10.2196/57962 (PMC11933748; doi:10.2196/57962)
Supplement: Multimedia Appendix 2 [file publichealth_v11i1e57962_app2.docx]

**Contents of Multimedia Appendix 2**

| **Multimedia Appendix 2** | | Page |
| --- | --- | --- |
| Table S1 | The trend in the average screen time (m/d) of adolescents in nuclear family group before and during COVID-19 pandemic, weighted mean (95% CI), in KYRBS. | P2–6 |
| Table S2 | The trend in the average screen time (m/d) of adolescents in living with relatives group before and during COVID-19 pandemic, weighted mean (95% CI), in KYRBS. | P7–11 |
| Table S3 | The trend in the average screen time (m/d) of adolescents in living alone group before and during COVID-19 pandemic, weighted mean (95% CI), in KYRBS. | P12–16 |
| Table S4 | The trend in the average screen time (m/d) of adolescents in orphanage group before and during COVID-19 pandemic, weighted mean (95% CI), in KYRBS. | P17–20 |
| Table S5 | Factors associated with weighted average screen time among adolescents in nuclear family group before and during COVID-19 pandemic in KYRBS. | P21–23 |
| Table S6 | Factors associated with weighted average screen time among adolescents in living with relatives group before and during COVID-19 pandemic in KYRBS. | P24–26 |
| Table S7 | Factors associated with weighted average screen time among adolescents in living alone group before and during COVID-19 pandemic in KYRBS. | P27–29 |
| Table S8 | Factors associated with weighted average screen time among adolescents in orphanage group before and during COVID-19 pandemic in KYRBS. | P30–32 |

**Table S1.** The trend in the average screen time (m/d) of adolescents in nuclear family group before and during COVID-19 pandemic, weighted mean (95% CI), in KYRBS.

|  | Pre-pandemic | | | | | | Pandemic | Trends in the pre-pandemic, β (95% CI) | Trends in the pandemic, β　(95% CI) | Trend differences, β_diff_ (95% CI) |
| --- | --- | --- | --- | --- | --- | --- | --- | --- | --- | --- |
| Year | 2008-2009 | 2010-2011 | 2012-2013 | 2014-2015 | 2016-2017 | 2018-2019 | 2020-2022 |  |  |  |
| Overall | 121.16 (119.80 to 122.52) | 112.18 (110.97 to 113.38) | 99.19 (97.75 to 100.63) | 100.71 (99.25 to 102.16) | 163.56 (160.82 to 166.30) | 144.34 (142.72 to 145.97) | 313.17 (310.45 to 315.89) | **6.88 (6.49 to 7.27)** | **168.83 (165.92 to 171.74)** | **156.20 (141.66 to 170.74)** |
| Sex | | | | | | | | | | |
| Men | 125.16 (123.27 to 127.05) | 115.05 (113.27 to 116.84) | 115.00 (113.08 to 116.92) | 115.90 (113.85 to 117.95) | 150.30 (147.25 to 153.35) | 135.05 (132.97 to 137.14) | 279.70 (276.58 to 282.82) | **4.08 (3.56 to 4.60)** | **144.64 (141.13 to 148.16)** | **134.18 (124.51 to 143.85)** |
| Women | 116.31 (114.61 to 118.00) | 108.88 (107.43 to 110.34) | 80.86 (79.34 to 82.39) | 84.39 (82.78 to 86.00) | 177.55 (173.15 to 181.96) | 152.97 (150.73 to 155.20) | 343.16 (339.84 to 346.49) | **10.05 (9.45 to 10.65)** | **190.19 (186.42 to 193.96)** | **170.52 (149.31 to 191.74)** |
| BMI group ^a^ | | | | | | | | | | |
| Underweight | 123.20 (119.93 to 126.47) | 114.11 (111.02 to 117.21) | 101.70 (97.74 to 105.66) | 105.28 (100.84 to 109.71) | 163.30 (156.96 to 169.63) | 142.14 (136.85 to 147.42) | 312.91 (306.95 to 318.87) | **5.68 (4.67 to 6.69)** | **170.77 (163.34 to 178.21)** | **166.83 (151.75 to 181.91)** |
| Normal | 118.65 (117.25 to 120.05) | 109.60 (108.33 to 110.88) | 95.31 (93.83 to 96.79) | 96.78 (95.27 to 98.29) | 160.21 (157.34 to 163.08) | 142.11 (140.24 to 143.98) | 307.95 (305.08 to 310.82) | **6.63 (6.22 to 7.05)** | **165.84 (162.69 to 168.98)** | **152.26 (138.09 to 166.42)** |
| Overweight | 126.12 (122.13 to 130.12) | 123.14 (119.02 to 127.25) | 108.58 (104.72 to 112.44) | 109.45 (105.58 to 113.32) | 169.54 (163.59 to 175.48) | 147.53 (143.22 to 151.84) | 309.02 (303.47 to 314.57) | **6.92 (5.91 to 7.92)** | **161.49 (154.95 to 168.02)** | **136.30 (119.46 to 153.13)** |
| Obese | 139.71 (134.56 to 144.85) | 126.91 (122.83 to 130.99) | 124.66 (120.11 to 129.21) | 122.20 (117.92 to 126.48) | 177.03 (171.44 to 182.63) | 154.03 (149.34 to 158.72) | 331.26 (325.97 to 336.54) | **6.74 (5.62 to 7.86)** | **177.23 (170.71 to 183.74)** | **155.03 (138.30 to 171.76)** |
| Grade | | | | | | | | | | |
| 7^th^ | 112.01 (109.22 to 114.79) | 104.52 (101.98 to 107.07) | 84.96 (82.03 to 87.90) | 90.39 (87.29 to 93.49) | 153.34 (148.64 to 158.04) | 147.00 (143.14 to 150.86) | 270.61 (266.01 to 275.20) | **7.76 (6.98 to 8.54)** | **123.60 (118.03 to 129.17)** | **117.60 (99.95 to 135.245)** |
| 8^th^ | 123.30 (120.76 to 125.83) | 116.31 (113.35 to 119.28) | 99.79 (96.77 to 102.81) | 107.62 (104.42 to 110.82) | 169.50 (164.34 to 174.66) | 157.51 (153.51 to 161.51) | 301.08 (296.51 to 305.65) | **8.14 (7.35 to 8.93)** | **143.57 (137.93 to 149.21)** | **131.82 (113.43 to 150.21)** |
| 9^th^ | 129.70 (126.94 to 132.47) | 120.16 (117.69 to 122.62) | 109.36 (106.22 to 112.49) | 108.71 (105.57 to 111.86) | 173.08 (168.21 to 177.95) | 156.87 (153.18 to 160.56) | 324.29 (319.57 to 329.00) | **6.99 (6.22 to 7.77)** | **167.42 (161.85 to 172.98)** | **153.78 (137.57 to 169.99)** |
| 10^th^ | 119.34 (116.57 to 122.11) | 113.30 (110.71 to 115.89) | 97.30 (94.38 to 100.21) | 97.70 (94.67 to 100.73) | 156.94 (151.99 to 161.90) | 142.86 (139.20 to 146.52) | 315.79 (310.57 to 321.01) | **6.24 (5.46 to 7.01)** | **172.93 (167.14 to 178.72)** | **154.71 (139.79 to 169.62)** |
| 11^th^ | 119.70 (116.95 to 122.46) | 109.82 (107.30 to 112.35) | 103.30 (100.42 to 106.19) | 103.13 (99.85 to 106.41) | 173.85 (168.44 to 179.25) | 137.29 (133.55 to 141.03) | 334.49 (328.88 to 340.11) | **8.01 (7.20 to 8.82)** | **197.20 (191.08 to 203.32)** | **182.72 (164.38 to 201.06)** |
| 12^th^ | 121.05 (118.00 to 124.10) | 108.13 (105.37 to 110.88) | 96.98 (94.08 to 99.87) | 94.90 (92.10 to 97.70) | 154.59 (149.24 to 159.93) | 130.71 (127.38 to 134.04) | 334.30 (328.47 to 340.14) | **5.02 (4.22 to 5.81)** | **203.60 (197.49 to 209.70)** | **187.91 (174.99 to 200.84)** |
| Region of residence | | | | | | | | | | |
| Urban | 121.09 (119.40 to 122.78) | 111.61 (110.16 to 113.07) | 99.60 (97.94 to 101.27) | 101.41 (99.63 to 103.19) | 161.59 (158.51 to 164.68) | 144.01 (142.03 to 145.99) | 308.90 (305.59 to 312.21) | **6.67 (6.21 to 7.13)** | **164.89 (161.36 to 168.42)** | **152.04 (137.63 to 166.45)** |
| Rural | 121.34 (119.14 to 123.53) | 113.60 (111.49 to 115.72) | 98.15 (95.31 to 100.99) | 98.99 (96.52 to 101.45) | 168.22 (162.61 to 173.83) | 145.12 (142.30 to 147.94) | 323.00 (318.28 to 327.72) | **7.37 (6.64 to 8.10)** | **177.88 (172.79 to 182.97)** | **161.67 (144.99 to 178.34)** |
| Smoking status | | | | | | | | | | |
| No | 118.36 (117.03 to 119.69) | 111.76 (110.53 to 112.99) | 98.94 (97.49 to 100.39) | 100.28 (98.81 to 101.75) | 162.67 (159.93 to 165.41) | 144.48 (142.83 to 146.12) | 311.67 (308.96 to 314.38) | **7.59 (7.20 to 7.98)** | **167.20 (164.29 to 170.11)** | **151.63 (135.71 to 167.54)** |
| Yes | 138.48 (134.96 to 142.00) | 116.41 (112.51 to 120.30) | 107.90 (101.14 to 114.66) | 111.60 (105.08 to 118.12) | 210.79 (193.11 to 228.47) | 137.51 (126.53 to 148.48) | 504.11 (485.14 to 523.09) | 1.55 (-0.32 to 3.42) | **366.61 (341.26 to 391.95)** | **351.33 (325.11 to 377.54)** |
| Alcoholic consumption | | | | | | | | | | |
| No | 116.98 (115.59 to 118.37) | 109.86 (108.53 to 111.19) | 97.17 (95.68 to 98.65) | 99.34 (97.86 to 100.82) | 155.96 (153.29 to 158.63) | 143.76 (142.05 to 145.47) | 302.35 (299.74 to 304.96) | **7.13 (6.74 to 7.53)** | **158.59 (155.72 to 161.45)** | **144.57 (129.54 to 159.60)** |
| Yes | 134.04 (131.61 to 136.48) | 120.32 (118.01 to 122.63) | 107.74 (104.89 to 110.59) | 107.01 (103.83 to 110.19) | 202.61 (196.57 to 208.65) | 147.22 (143.35 to 151.08) | 399.24 (392.65 to 405.84) | **7.28 (6.46 to 8.09)** | **252.02 (244.92 to 259.13)** | **237.06 (219.68 to 254.44)** |
| School performance ^b^ | | | | | | | | | | |
| Low | 145.16 (141.57 to 148.75) | 133.28 (130.01 to 136.55) | 113.28 (109.91 to 116.64) | 119.47 (115.41 to 123.53) | 212.54 (204.62 to 220.46) | 163.23 (157.47 to 169.00) | 415.67 (408.36 to 422.98) | **8.11 (6.96 to 9.27)** | **252.44 (243.80 to 261.07)** | **233.25 (213.14 to 253.36)** |
| Middle-low | 130.61 (128.54 to 132.67) | 121.18 (119.23 to 123.12) | 106.44 (104.10 to 108.78) | 108.84 (106.39 to 111.29) | 183.92 (179.33 to 188.51) | 154.08 (150.95 to 157.21) | 360.36 (356.30 to 364.43) | **7.63 (6.98 to 8.29)** | **206.28 (201.58 to 210.99)** | **193.75 (176.84 to 210.66)** |
| Middle | 116.19 (114.36 to 118.02) | 107.44 (105.63 to 109.24) | 95.83 (93.77 to 97.90) | 97.90 (95.75 to 100.05) | 159.24 (155.88 to 162.60) | 145.21 (142.73 to 147.70) | 309.29 (305.98 to 312.59) | **7.86 (7.35 to 8.38)** | **164.08 (160.28 to 167.87)** | **150.70 (133.84 to 167.57)** |
| Middle-high | 112.72 (110.75 to 114.69) | 105.13 (103.31 to 106.95) | 93.68 (91.53 to 95.82) | 94.91 (92.80 to 97.03) | 146.12 (142.84 to 149.41) | 137.31 (134.79 to 139.84) | 274.07 (270.86 to 277.28) | **6.31 (5.79 to 6.84)** | **136.76 (132.99 to 140.53)** | **126.11 (112.17 to 140.04)** |
| High | 98.98 (95.87 to 102.09) | 92.52 (89.75 to 95.30) | 84.24 (80.88 to 87.60) | 83.80 (80.64 to 86.96) | 129.15 (124.56 to 133.74) | 122.48 (118.98 to 125.98) | 238.48 (234.14 to 242.82) | **5.95 (5.18 to 6.73)** | **116.00 (110.81 to 121.18)** | **108.78 (94.61 to 122.95)** |
| Sexual experience | | | | | | | | | | |
| No | 119.70 (118.28 to 121.12) | 111.43 (110.18 to 112.68) | 98.84 (97.38 to 100.30) | 100.84 (99.37 to 102.31) | 161.60 (158.86 to 164.35) | 145.20 (143.55 to 146.84) | 308.53 (305.89 to 311.18) | **7.50 (7.10 to 7.89)** | **163.34 (160.48 to 166.20)** | **150.29 (134.55 to 166.02)** |
| Yes | 126.64 (123.98 to 129.30) | 115.17 (112.69 to 117.65) | 101.95 (98.36 to 105.54) | 98.29 (92.69 to 103.90) | 201.61 (190.11 to 213.11) | 130.24 (123.59 to 136.90) | 406.36 (396.04 to 416.69) | **4.10 (2.84 to 5.36)** | **276.12 (264.49 to 287.75)** | **259.27 (243.59 to 274.95)** |
| Physical activity frequency ^c^ | | | | | | | | | | |
| Lower activity | 123.30 (121.77 to 124.83) | 114.18 (112.76 to 115.61) | 99.03 (97.29 to 100.78) | 99.80 (98.10 to 101.51) | 168.35 (164.82 to 171.89) | 150.10 (148.08 to 152.12) | 320.68 (317.69 to 323.67) | **7.40 (6.92 to 7.87)** | **170.58 (167.27 to 173.89)** | **155.11 (139.33 to 170.90)** |
| Moderate activity | 119.18 (116.99 to 121.36) | 110.81 (108.81 to 112.80) | 99.92 (97.78 to 102.06) | 104.63 (102.32 to 106.93) | 157.09 (153.63 to 160.55) | 139.91 (137.08 to 142.75) | 297.45 (293.04 to 301.85) | **6.30 (5.71 to 6.89)** | **157.53 (152.70 to 162.36)** | **145.87 (131.53 to 160.20)** |
| Higher activity | 115.22 (112.37 to 118.06) | 105.72 (103.00 to 108.43) | 98.45 (95.42 to 101.48) | 97.35 (94.28 to 100.42) | 156.25 (151.66 to 160.83) | 128.22 (124.71 to 131.74) | 294.74 (290.30 to 299.17) | **5.96 (5.20 to 6.72)** | **166.52 (161.21 to 171.82)** | **153.62 (139.42 to 167.82)** |
| Sadness and despair | | | | | | | | | | |
| No | 118.81 (117.27 to 120.36) | 110.43 (109.03 to 111.84) | 99.96 (98.37 to 101.56) | 99.94 (98.37 to 101.52) | 157.31 (154.51 to 160.11) | 139.58 (137.79 to 141.37) | 301.12 (298.38 to 303.87) | **6.58 (6.16 to 7.00)** | **161.55 (158.54 to 164.55)** | **146.46 (132.41 to 160.50)** |
| Yes | 124.85 (123.04 to 126.65) | 115.27 (113.55 to 116.99) | 97.51 (95.42 to 99.60) | 102.92 (100.49 to 105.35) | 181.64 (177.35 to 185.94) | 156.50 (153.44 to 159.56) | 346.11 (341.92 to 350.29) | **8.28 (7.67 to 8.88)** | **189.61 (184.84 to 194.37)** | **175.45 (157.42 to 193.49)** |
| Highest educational level of parents | | | | | | | | | | |
| High school or lower | 108.38 (106.80 to 109.96) | 102.48 (100.99 to 103.96) | 92.44 (90.80 to 94.08) | 95.48 (93.88 to 97.08) | 151.68 (148.84 to 154.52) | 140.15 (138.23 to 142.08) | 290.29 (287.62 to 292.97) | **8.55 (8.13 to 8.97)** | **150.14 (147.12 to 153.16)** | **133.99 (116.14 to 151.83)** |
| College or higher | 131.47 (129.82 to 133.12) | 121.31 (119.77 to 122.85) | 105.91 (103.98 to 107.83) | 107.02 (104.95 to 109.09) | 179.75 (176.00 to 183.50) | 151.13 (148.60 to 153.65) | 358.28 (354.50 to 362.05) | **6.26 (5.71 to 6.82)** | **207.15 (202.97 to 211.33)** | **193.14 (179.15 to 207.14)** |

Abbreviations: BMI, body mass index (calculated as weight in kilograms divided by height in meters squared); CI, confidence interval; KYRBS, Korea Youth Risk Behavior Web-Based Survey.

Numbers in bold indicate a significant difference (*P* < 0.05).

^a^ BMI was divided into four groups according to the 2017 Korean National Growth Charts: underweight (<5 percentile), normal (5 to 84 percentile), overweight (85 to 94 percentile), and obese (≥95 percentile).

^b^ School performance was divided into five groups: low (<20 percentile), middle-low (20 to 39 percentile), middle (40 to 59 percentile), middle-high (60 to 79 percentile), and high (≥80 percentile).

^c^ Physical activity frequency was divided into the three groups based on the engagement in vigorous aerobic and resistance trainings more than three days per week: lower activity (neither activity is done for more than three days per week), moderate activity (either one activity), and higher activity (both activities).

**Table S2.** The trend in the average screen time (m/d) of adolescents in living with relatives group before and during COVID-19 pandemic, weighted mean (95% CI), in KYRBS.

|  | Pre-pandemic | | | | | | Pandemic | Trends in the pre-pandemic, β (95% CI) | Trends in the pandemic, β　(95% CI) | Trend differences, β_diff_ (95% CI) |
| --- | --- | --- | --- | --- | --- | --- | --- | --- | --- | --- |
| Year | 2008-2009 | 2010-2011 | 2012-2013 | 2014-2015 | 2016-2017 | 2018-2019 | 2020-2022 |  |  |  |
| Overall | 134.68 (129.12 to 140.25) | 127.36 (121.20 to 133.51) | 119.10 (112.92 to 125.28) | 123.52 (115.98 to 131.05) | 201.98 (189.44 to 214.51) | 157.75 (148.20 to 167.31) | 353.48 (340.34 to 366.62) | **9.24 (7.30 to 11.17)** | **195.73 (179.58 to 211.87)** | **183.10 (155.89 to 210.30)** |
| Sex | | | | | | | | | | |
| Male | 143.70 (137.20 to 150.21) | 130.00 (121.82 to 138.18) | 138.02 (129.86 to 146.17) | 135.14 (125.97 to 144.31) | 180.45 (165.31 to 195.60) | 151.88 (139.24 to 164.52) | 299.62 (283.62 to 315.61) | **5.15 (2.55 to 7.75)** | **147.74 (125.63 to 169.84)** | **137.27 (110.46 to 164.09)** |
| Female | 121.90 (114.85 to 128.96) | 124.15 (116.82 to 131.48) | 95.82 (87.82 to 103.82) | 111.09 (100.08 to 122.10) | 227.26 (208.78 to 245.73) | 164.69 (153.41 to 175.97) | 409.19 (392.61 to 425.78) | **14.59 (11.62 to 17.55)** | **244.50 (221.79 to 267.21)** | **224.83 (183.62 to 266.05)** |
| BMI group ^a^ | | | | | | | | | | |
| Underweight | 150.09 (136.32 to 163.85) | 131.57 (114.51 to 148.64) | 125.80 (111.37 to 140.24) | 137.01 (118.09 to 155.93) | 156.92 (135.36 to 178.47) | 148.06 (125.18 to 170.94) | 394.65 (372.87 to 416.44) | 0.62 (-5.76 to 7.01) | **246.59 (186.69 to 306.50)** | **242.65 (181.20 to 304.11)** |
| Normal | 135.20 (129.28 to 141.11) | 130.61 (123.23 to 137.99) | 117.52 (110.48 to 124.55) | 119.56 (111.29 to 127.83) | 207.12 (191.73 to 222.52) | 162.70 (150.73 to 174.66) | 352.62 (338.18 to 367.06) | **9.53 (7.18 to 11.88)** | **189.92 (170.07 to 209.78)** | **176.34 (145.74 to 206.94)** |
| Overweight | 149.51 (137.25 to 161.77) | 129.19 (114.13 to 144.24) | 124.59 (107.14 to 142.03) | 140.67 (124.24 to 157.09) | 216.61 (186.70 to 246.52) | 160.52 (143.86 to 177.19) | 334.33 (303.22 to 365.44) | **9.76 (3.30 to 16.22)** | **173.81 (125.48 to 222.14)** | **148.62 (90.76 to 206.47)** |
| Obese | 151.85 (135.56 to 168.14) | 114.41 (100.97 to 127.85) | 149.20 (138.36 to 160.03) | 164.60 (144.92 to 184.28) | 209.88 (190.32 to 229.45) | 177.85 (156.94 to 198.77) | 380.94 (358.37 to 403.51) | **12.96 (6.20 to 19.72)** | **203.09 (160.07 to 246.10)** | **180.89 (123.06 to 238.73)** |
| Grade | | | | | | | | | | |
| 7^th^ | 122.86 (109.66 to 136.07) | 116.34 (101.01 to 131.68) | 106.11 (93.10 to 119.12) | 116.02 (95.10 to 136.94) | 186.28 (161.80 to 210.75) | 153.77 (132.49 to 175.06) | 324.43 (299.61 to 349.25) | **9.10 (4.57 to 13.64)** | **170.65 (136.60 to 204.71)** | **164.65 (121.36 to 207.94)** |
| 8^th^ | 129.72 (118.49 to 140.95) | 130.60 (115.28 to 145.93) | 123.08 (106.73 to 139.43) | 114.07 (97.79 to 130.34) | 198.85 (173.25 to 224.45) | 179.72 (157.65 to 201.79) | 371.86 (344.37 to 399.35) | **10.05 (5.70 to 14.40)** | **192.14 (154.91 to 229.38)** | **180.40 (133.67 to 227.13)** |
| 9^th^ | 142.30 (129.42 to 155.18) | 127.38 (113.43 to 141.34) | 136.44 (120.10 to 152.78) | 119.80 (101.30 to 138.31) | 210.76 (185.20 to 236.33) | 164.55 (141.11 to 187.99) | 347.27 (314.09 to 380.44) | **8.90 (4.40 to 13.40)** | **182.72 (141.99 to 223.44)** | **169.08 (120.62 to 217.54)** |
| 10^th^ | 134.83 (124.65 to 145.01) | 122.36 (111.35 to 133.38) | 115.05 (101.51 to 128.58) | 125.88 (107.37 to 144.39) | 208.15 (185.62 to 230.67) | 164.94 (150.45 to 179.44) | 361.87 (334.81 to 388.93) | **11.65 (7.30 to 16.00)** | **196.93 (156.83 to 237.03)** | **178.71 (127.81 to 229.61)** |
| 11^th^ | 140.17 (127.76 to 152.57) | 132.53 (121.75 to 143.32) | 119.92 (104.50 to 135.35) | 140.75 (124.02 to 157.48) | 216.05 (190.26 to 241.83) | 159.49 (138.84 to 180.14) | 369.42 (341.86 to 396.98) | **10.59 (5.89 to 15.29)** | **209.93 (168.02 to 251.85)** | **195.45 (143.92 to 246.98)** |
| 12^th^ | 138.88 (129.05 to 148.71) | 133.46 (121.11 to 145.82) | 112.35 (102.26 to 122.43) | 119.81 (103.31 to 136.31) | 189.84 (163.13 to 216.56) | 135.73 (119.10 to 152.37) | 347.52 (323.35 to 371.68) | **5.14 (0.35 to 9.93)** | **211.79 (172.74 to 250.83)** | **196.10 (152.48 to 239.72)** |
| Region of residence | | | | | | | | | | |
| Urban | 129.08 (121.50 to 136.66) | 122.74 (114.13 to 131.35) | 115.20 (107.39 to 123.02) | 123.53 (113.35 to 133.71) | 209.20 (191.58 to 226.82) | 159.49 (146.45 to 172.54) | 349.12 (332.39 to 365.85) | **11.38 (8.74 to 14.02)** | **189.63 (168.92 to 210.33)** | **176.77 (142.36 to 211.18)** |
| Rural | 143.09 (135.08 to 151.10) | 133.44 (124.72 to 142.17) | 123.74 (113.86 to 133.63) | 123.50 (112.25 to 134.74) | 192.30 (174.98 to 209.62) | 155.19 (141.34 to 169.04) | 360.75 (339.50 to 382.01) | **6.15 (3.35 to 8.96)** | **205.57 (179.59 to 231.54)** | **189.35 (158.00 to 220.70)** |
| Smoking status | | | | | | | | | | |
| No | 130.92 (125.09 to 136.75) | 128.53 (122.15 to 134.92) | 120.43 (114.06 to 126.81) | 124.66 (116.78 to 132.54) | 203.13 (190.59 to 215.67) | 161.81 (152.19 to 171.43) | 352.58 (339.37 to 365.79) | **10.82 (8.81 to 12.82)** | **190.77 (174.62 to 206.93)** | **175.20 (145.33 to 205.08)** |
| Yes | 147.20 (136.65 to 157.74) | 120.77 (108.28 to 133.26) | 95.03 (80.35 to 109.71) | 107.64 (91.03 to 124.24) | 182.64 (165.21 to 200.07) | 86.59 (66.33 to 106.85) | 406.08 (-103.22 to 915.38) | -5.54 (-11.77 to 0.69) | **319.49 (164.43 to 474.54)** | **304.21 (149.14 to 459.27)** |
| Alcoholic consumption | | | | | | | | | | |
| No | 131.04 (124.91 to 137.16) | 126.03 (119.43 to 132.63) | 125.39 (118.01 to 132.77) | 126.12 (117.80 to 134.43) | 191.10 (179.10 to 203.09) | 160.24 (149.48 to 170.99) | 342.80 (329.66 to 355.95) | **9.44 (7.37 to 11.50)** | **182.57 (165.33 to 199.81)** | **168.55 (140.17 to 196.94)** |
| Yes | 143.16 (132.68 to 153.63) | 130.34 (119.88 to 140.80) | 101.33 (91.23 to 111.43) | 115.25 (102.62 to 127.87) | 237.97 (209.50 to 266.45) | 149.95 (130.88 to 169.02) | 400.23 (369.52 to 430.93) | **8.92 (4.59 to 13.24)** | **250.28 (208.50 to 292.06)** | **235.31 (186.13 to 284.49)** |
| School performance ^b^ | | | | | | | | | | |
| Low | 153.35 (142.05 to 164.64) | 134.49 (123.40 to 145.58) | 122.52 (110.84 to 134.21) | 130.96 (115.24 to 146.67) | 218.74 (190.26 to 247.23) | 160.41 (143.55 to 177.28) | 446.65 (414.37 to 478.93) | **7.46 (2.63 to 12.30)** | **286.24 (241.94 to 330.54)** | **267.05 (216.61 to 317.49)** |
| Middle-low | 137.76 (129.14 to 146.38) | 127.34 (117.99 to 136.68) | 122.64 (112.29 to 133.00) | 129.72 (117.29 to 142.15) | 223.77 (203.33 to 244.21) | 168.65 (152.78 to 184.51) | 414.06 (392.69 to 435.42) | **11.84 (8.40 to 15.29)** | **245.41 (215.80 to 275.03)** | **232.88 (190.75 to 275.01)** |
| Middle | 123.35 (114.58 to 132.12) | 122.03 (111.77 to 132.28) | 128.93 (117.53 to 140.33) | 110.49 (99.53 to 121.44) | 209.87 (192.01 to 227.72) | 152.86 (138.20 to 167.51) | 327.40 (311.39 to 343.40) | **11.43 (7.96 to 14.91)** | **174.54 (147.47 to 201.61)** | **161.17 (121.34 to 201.00)** |
| Middle-high | 123.20 (111.71 to 134.68) | 127.23 (115.30 to 139.15) | 108.20 (97.54 to 118.86) | 123.47 (109.22 to 137.72) | 170.77 (151.54 to 189.99) | 157.91 (141.34 to 174.47) | 282.44 (266.80 to 298.08) | **8.49 (4.21 to 12.77)** | **124.54 (93.78 to 155.29)** | **113.88 (74.23 to 153.54)** |
| High | 118.73 (105.41 to 132.05) | 119.01 (105.68 to 132.34) | 86.43 (75.34 to 97.53) | 117.82 (99.54 to 136.10) | 139.31 (126.10 to 152.51) | 130.04 (115.29 to 144.80) | 204.24 (173.95 to 234.53) | 3.90 (-2.36 to 10.16) | **74.19 (18.51 to 129.88)** | **66.98 (7.84 to 126.12)** |
| Sexual experience | | | | | | | | | | |
| No | 133.12 (127.17 to 139.08) | 126.43 (119.76 to 133.10) | 122.96 (116.13 to 129.79) | 127.84 (119.84 to 135.83) | 199.26 (187.12 to 211.40) | 160.54 (150.79 to 170.30) | 348.64 (335.43 to 361.86) | **10.20 (8.19 to 12.21)** | **188.10 (171.74 to 204.46)** | **175.05 (146.07 to 204.03)** |
| Yes | 138.55 (127.85 to 149.24) | 129.48 (117.36 to 141.60) | 104.13 (92.33 to 115.93) | 89.94 (73.89 to 106.00) | 220.98 (190.60 to 251.35) | 142.19 (121.96 to 162.41) | 390.69 (360.28 to 421.10) | 4.51 (-1.07 to 10.08) | **248.50 (188.71 to 308.29)** | **231.65 (168.68 to 294.62)** |
| Physical activity frequency ^c^ | | | | | | | | | | |
| Lower activity | 137.06 (129.75 to 144.37) | 135.17 (127.32 to 143.02) | 120.34 (112.51 to 128.16) | 124.90 (115.66 to 134.15) | 204.00 (189.82 to 218.18) | 173.58 (160.89 to 186.26) | 366.41 (351.21 to 381.60) | **10.14 (7.64 to 12.63)** | **192.83 (172.49 to 213.17)** | **177.36 (145.32 to 209.41)** |
| Moderate activity | 132.83 (123.98 to 141.69) | 115.27 (105.71 to 124.83) | 120.14 (109.74 to 130.54) | 132.04 (118.65 to 145.43) | 199.78 (181.72 to 217.85) | 149.19 (134.52 to 163.86) | 328.92 (310.43 to 347.42) | **9.73 (6.23 to 13.22)** | **179.73 (142.85 to 216.61)** | **168.06 (122.99 to 213.14)** |
| Higher activity | 128.91 (116.78 to 141.04) | 116.09 (100.45 to 131.72) | 112.15 (99.29 to 125.01) | 103.99 (93.57 to 114.40) | 198.30 (173.58 to 223.03) | 120.57 (108.40 to 132.74) | 324.77 (300.61 to 348.92) | **5.83 (1.33 to 10.34)** | **204.19 (166.61 to 241.77)** | **191.30 (148.60 to 233.99)** |
| Sadness and despair | | | | | | | | | | |
| No | 130.80 (123.61 to 137.99) | 127.90 (120.27 to 135.53) | 123.36 (116.44 to 130.28) | 126.13 (117.18 to 135.08) | 199.06 (185.63 to 212.49) | 153.50 (142.47 to 164.53) | 341.36 (325.94 to 356.77) | **9.78 (7.39 to 12.16)** | **187.85 (168.45 to 207.26)** | **172.77 (142.02 to 203.51)** |
| Yes | 139.27 (130.58 to 147.96) | 126.70 (116.64 to 136.77) | 112.27 (102.22 to 122.33) | 118.62 (107.16 to 130.09) | 208.13 (187.82 to 228.44) | 164.82 (149.65 to 180.00) | 377.36 (355.19 to 399.54) | **8.48 (5.17 to 11.79)** | **212.54 (183.60 to 241.49)** | **198.39 (161.35 to 235.43)** |
| Highest educational level of parents | | | | | | | | | | |
| High school or lower | 133.02 (122.22 to 143.81) | 132.70 (121.79 to 143.62) | 114.28 (104.16 to 124.40) | 127.89 (115.58 to 140.20) | 207.44 (188.21 to 226.67) | 172.70 (157.92 to 187.48) | 348.42 (325.94 to 370.91) | **12.43 (8.70 to 16.15)** | **175.72 (145.45 to 206.00)** | **159.57 (115.77 to 203.38)** |
| College or higher | 135.15 (129.10 to 141.21) | 125.89 (118.95 to 132.82) | 120.50 (113.38 to 127.63) | 122.25 (113.43 to 131.08) | 199.84 (184.41 to 215.27) | 153.08 (141.89 to 164.28) | 355.12 (340.05 to 370.18) | **8.18 (5.91 to 10.46)** | **202.03 (182.91 to 221.16)** | **188.03 (160.00 to 216.06)** |

Abbreviations: BMI, body mass index (calculated as weight in kilograms divided by height in meters squared); CI, confidence interval; KYRBS, Korea Youth Risk Behavior Web-Based Survey.

Numbers in bold indicate a significant difference (*P* < 0.05).

^a^ BMI was divided into four groups according to the 2017 Korean National Growth Charts: underweight (<5 percentile), normal (5 to 84 percentile), overweight (85 to 94 percentile), and obese (≥95 percentile).

^b^ School performance was divided into five groups: low (<20 percentile), middle-low (20 to 39 percentile), middle (40 to 59 percentile), middle-high (60 to 79 percentile), and high (≥80 percentile).

^c^ Physical activity frequency was divided into the three groups based on the engagement in vigorous aerobic and resistance trainings more than three days per week: lower activity (neither activity is done for more than three days per week), moderate activity (either one activity), and higher activity (both activities).

**Table S3.** The trend in the average screen time (m/d) of adolescents in living alone group before and during COVID-19 pandemic, weighted mean (95% CI), in KYRBS.

|  | Pre-pandemic | | | | | | Pandemic | Trends in the pre-pandemic, β (95% CI) | Trends in the pandemic, β　(95% CI) | Trend differences, β_diff_ (95% CI) |
| --- | --- | --- | --- | --- | --- | --- | --- | --- | --- | --- |
| Year | 2008-2009 | 2010-2011 | 2012-2013 | 2014-2015 | 2016-2017 | 2018-2019 | 2020-2022 |  |  |  |
| Overall | 111.39 (100.87 to 121.91) | 111.67 (100.79 to 122.54) | 92.92 (79.03 to 106.81) | 82.85 (71.40 to 94.29) | 150.79 (128.72 to 172.85) | 122.58 (112.99 to 132.17) | 257.47 (235.46 to 279.48) | **4.90 (1.96 to 7.84)** | **134.89 (111.26 to 158.52)** | **122.26 (94.07 to 150.44)** |
| Sex | | | | | | | | | | |
| Male | 107.29 (95.04 to 119.55) | 115.41 (102.09 to 128.73) | 90.05 (76.43 to 103.67) | 85.15 (74.20 to 96.09) | 119.68 (98.29 to 141.06) | 113.73 (102.26 to 125.20) | 239.62 (213.79 to 265.45) | 1.67 (-2.25 to 5.58) | **125.89 (96.87 to 154.91)** | **115.43 (84.41 to 146.44)** |
| Female | 116.88 (107.03 to 126.73) | 106.78 (94.57 to 118.99) | 96.61 (83.31 to 109.92) | 80.00 (63.37 to 96.63) | 190.02 (164.94 to 215.09) | 134.54 (122.85 to 146.24) | 293.95 (271.87 to 316.04) | **9.21 (5.00 to 13.42)** | **159.41 (131.53 to 187.29)** | **139.74 (101.41 to 178.07)** |
| BMI group ^a^ | | | | | | | | | | |
| Underweight | 120.32 (103.53 to 137.11) | 117.11 (97.74 to 136.48) | 134.92 (100.56 to 169.28) | 108.84 (85.50 to 132.17) | 150.42 (109.80 to 191.04) | 134.34 (99.04 to 169.64) | 246.13 (218.06 to 274.19) | 4.10 (-6.03 to 14.24) | **111.79 (59.71 to 163.86)** | **107.85 (48.77 to 166.93)** |
| Normal | 102.74 (93.55 to 111.93) | 112.62 (100.90 to 124.34) | 74.43 (63.20 to 85.65) | 81.31 (69.28 to 93.34) | 145.78 (120.46 to 171.10) | 116.75 (106.61 to 126.89) | 251.37 (228.70 to 274.03) | **4.92 (1.62 to 8.21)** | **134.62 (109.63 to 159.61)** | **121.04 (91.31 to 150.76)** |
| Overweight | 133.42 (-19.37 to 286.21) | 123.51 | 93.76 (55.87 to 131.64) | 79.35 (71.53 to 87.16) | 113.08 (83.85 to 142.32) | 119.41 (103.53 to 135.28) | 264.54 (237.82 to 291.27) | -1.27 (-10.55 to 8.00) | **145.14 (97.48 to 192.80)** | **119.95 (69.77 to 170.12)** |
| Obese | 152.41 (139.78 to 165.04) | 149.74 (123.93 to 175.56) | 190.8 | 112.81 (96.54 to 129.09) | 189.23 (175.44 to 203.02) | 171.97 (153.15 to 190.80) | 294.81 (259.82 to 329.80) | 5.07 (-5.70 to 15.84) | **122.83 (71.13 to 174.53)** | **100.64 (40.33 to 160.95)** |
| Grade | | | | | | | | | | |
| 7^th^ | 119.75 | 70.12 (55.75 to 84.49) | 126.82 (96.61 to 157.03) | 86.65 (62.14 to 111.16) | 89.75 | 68.68 (50.65 to 86.70) | 257.40 (148.37 to 366.44) | -5.79 (-21.98 to 10.39) | **188.73 (56.88 to 320.57)** | **182.72 (49.32 to 316.13)** |
| 8^th^ | 105.33 (101.20 to 109.46) | 90.01 (76.56 to 103.47) | 82.65 (55.28 to 110.01) | 26.95 (23.82 to 30.08) | 236.14 (65.51 to 406.78) | 103.04 (80.69 to 125.39) | 240.01 (183.81 to 296.20) | 7.53 (-10.03 to 25.10) | **136.96 (64.08 to 209.85)** | **125.22 (37.28 to 213.15)** |
| 9^th^ | 135.08 (116.26 to 153.89) | 114.84 (90.06 to 139.62) | 121.83 (66.79 to 176.86) | 88.81 (34.69 to 142.93) | 83.06 (68.83 to 97.29) | 95.36 (59.22 to 131.50) | 265.16 (223.71 to 306.61) | -8.90 (-21.66 to 3.86) | **169.80 (104.86 to 234.74)** | **156.17 (90.79 to 221.54)** |
| 10^th^ | 111.49 (97.53 to 125.45) | 102.53 (88.16 to 116.90) | 57.75 (42.79 to 72.72) | 81.53 (59.93 to 103.13) | 113.31 (85.97 to 140.66) | 140.80 (125.31 to 156.29) | 257.52 (230.64 to 284.40) | **8.24 (2.91 to 13.57)** | **116.72 (84.45 to 148.99)** | **98.50 (56.68 to 140.32)** |
| 11^th^ | 104.26 (88.60 to 119.91) | 123.26 (105.24 to 141.27) | 107.85 (86.30 to 129.40) | 98.13 (84.23 to 112.04) | 193.70 (157.85 to 229.55) | 124.45 (108.88 to 140.02) | 259.83 (233.66 to 285.99) | **7.18 (2.08 to 12.27)** | **135.38 (103.22 to 167.53)** | **120.90 (80.74 to 161.05)** |
| 12^th^ | 112.10 (99.77 to 124.43) | 115.08 (103.61 to 126.56) | 95.55 (81.07 to 110.04) | 80.06 (68.25 to 91.86) | 147.70 (123.35 to 172.06) | 115.36 (101.57 to 129.14) | 256.26 (233.04 to 279.48) | 2.94 (-1.38 to 7.26) | **140.91 (108.20 to 173.62)** | **125.22 (89.55 to 160.90)** |
| Region of residence | | | | | | | | | | |
| Urban | 119.93 (102.58 to 137.27) | 110.84 (97.31 to 124.37) | 90.78 (70.86 to 110.71) | 94.08 (76.88 to 111.28) | 167.45 (137.73 to 197.18) | 123.66 (107.85 to 139.48) | 255.36 (227.47 to 283.24) | **5.94 (1.18 to 10.69)** | **131.70 (99.71 to 163.68)** | **118.84 (80.60 to 157.08)** |
| Rural | 104.93 (92.20 to 117.65) | 112.28 (96.05 to 128.51) | 95.26 (75.69 to 114.84) | 74.62 (60.01 to 89.24) | 130.16 (101.21 to 159.11) | 121.59 (110.21 to 132.97) | 259.40 (225.53 to 293.26) | **3.71 (0.20 to 7.22)** | **137.81 (103.05 to 172.57)** | **121.60 (84.07 to 159.12)** |
| Smoking status | | | | | | | | | | |
| No | 109.25 (97.86 to 120.63) | 106.19 (94.81 to 117.56) | 84.38 (71.55 to 97.20) | 80.28 (70.16 to 90.41) | 149.85 (126.70 to 173.01) | 124.16 (114.55 to 133.78) | 250.66 (228.85 to 272.46) | **6.64 (3.56 to 9.71)** | **126.49 (102.94 to 150.05)** | **110.92 (80.64 to 141.20)** |
| Yes | 116.82 (104.32 to 129.32) | 136.45 (113.09 to 159.81) | 180.69 (109.75 to 251.63) | 111.82 (85.12 to 138.53) | 160.25 (116.52 to 203.97) | 92.78 (72.66 to 112.91) | 462.97 (370.54 to 555.40) | -0.01 (-11.06 to 11.05) | **370.19 (235.28 to 505.10)** | **354.91 (218.27 to 491.54)** |
| Alcoholic consumption | | | | | | | | | | |
| No | 94.12 (82.14 to 106.09) | 102.92 (89.34 to 116.49) | 74.48 (61.17 to 87.80) | 65.78 (56.53 to 75.02) | 121.42 (100.76 to 142.07) | 124.19 (112.89 to 135.49) | 239.31 (217.66 to 260.95) | **7.28 (4.00 to 10.56)** | **115.12 (90.77 to 139.47)** | **101.10 (69.15 to 133.06)** |
| Yes | 135.80 (122.88 to 148.72) | 124.44 (112.21 to 136.68) | 123.22 (101.44 to 145.00) | 123.88 (104.02 to 143.74) | 206.34 (181.24 to 231.43) | 117.99 (100.90 to 135.09) | 318.67 (296.52 to 340.82) | 3.89 (-1.46 to 9.24) | **200.68 (160.39 to 240.97)** | **185.71 (141.54 to 229.88)** |
| School performance ^b^ | | | | | | | | | | |
| Low | 145.83 (132.81 to 158.84) | 157.15 (133.44 to 180.87) | 135.89 (109.67 to 162.11) | 96.48 (80.31 to 112.66) | 173.40 (125.51 to 221.30) | 121.89 (99.61 to 144.18) | 379.56 (344.32 to 414.79) | -2.92 (-11.21 to 5.37) | **257.66 (204.83 to 310.49)** | **238.47 (184.60 to 292.34)** |
| Middle-low | 108.95 (99.59 to 118.31) | 116.42 (101.49 to 131.36) | 104.63 (80.02 to 129.24) | 88.81 (68.54 to 109.09) | 173.53 (148.23 to 198.84) | 124.47 (109.69 to 139.25) | 297.67 (277.86 to 317.49) | **6.12 (0.52 to 11.71)** | **173.20 (141.34 to 205.07)** | **160.67 (121.40 to 199.94)** |
| Middle | 103.34 (91.31 to 115.38) | 104.82 (89.72 to 119.91) | 80.88 (69.94 to 91.82) | 75.61 (66.45 to 84.76) | 147.23 (125.59 to 168.87) | 124.37 (110.11 to 138.63) | 234.04 (212.21 to 255.86) | **6.75 (1.90 to 11.60)** | **109.67 (81.35 to 137.98)** | **96.29 (59.98 to 132.61)** |
| Middle-high | 97.35 (83.60 to 111.09) | 85.87 (78.07 to 93.67) | 70.76 (63.64 to 77.89) | 70.90 (58.65 to 83.15) | 131.51 (108.08 to 154.94) | 116.90 (102.93 to 130.86) | 208.11 (179.57 to 236.65) | **7.76 (3.31 to 12.21)** | **91.22 (58.59 to 123.84)** | **80.56 (40.11 to 121.02)** |
| High | 98.31 (87.22 to 109.41) | 90.63 (66.02 to 115.24) | 71.15 (62.19 to 80.11) | 91.06 (76.01 to 106.11) | 134.11 (99.14 to 169.07) | 126.41 (99.98 to 152.84) | 215.66 (193.68 to 237.64) | **8.79 (0.42 to 17.17)** | **89.25 (44.82 to 133.68)** | **82.03 (26.31 to 137.76)** |
| Sexual experience | | | | | | | | | | |
| No | 100.94 (89.72 to 112.16) | 106.27 (95.21 to 117.32) | 88.46 (76.44 to 100.47) | 75.23 (66.51 to 83.94) | 152.33 (126.33 to 178.34) | 126.22 (116.57 to 135.88) | 249.83 (227.46 to 272.21) | **8.20 (4.97 to 11.43)** | **123.61 (99.32 to 147.91)** | **110.56 (77.51 to 143.62)** |
| Yes | 123.48 (110.48 to 136.48) | 118.29 (102.41 to 134.18) | 102.53 (88.04 to 117.03) | 120.54 (96.69 to 144.39) | 145.44 (120.87 to 170.01) | 101.50 (78.26 to 124.74) | 298.71 (265.52 to 331.91) | -0.57 (-6.58 to 5.44) | **197.21 (147.78 to 246.64)** | **180.36 (129.80 to 230.93)** |
| Physical activity frequency ^c^ | | | | | | | | | | |
| Lower activity | 112.16 (100.51 to 123.81) | 116.39 (104.67 to 128.10) | 91.82 (76.20 to 107.44) | 82.71 (71.06 to 94.36) | 160.07 (133.69 to 186.45) | 138.37 (126.64 to 150.11) | 263.79 (237.52 to 290.07) | **7.67 (3.99 to 11.35)** | **125.42 (96.49 to 154.35)** | **109.95 (73.46 to 146.45)** |
| Moderate activity | 117.84 (102.48 to 133.19) | 100.47 (83.47 to 117.47) | 93.09 (73.29 to 112.89) | 80.96 (68.65 to 93.26) | 121.83 (96.55 to 147.12) | 113.52 (99.26 to 127.78) | 228.62 (204.08 to 253.15) | 1.40 (-4.43 to 7.23) | **115.10 (81.72 to 148.48)** | **103.43 (67.17 to 139.70)** |
| Higher activity | 97.67 (89.28 to 106.05) | 111.86 (101.94 to 121.78) | 96.37 (66.37 to 126.38) | 86.30 (72.64 to 99.96) | 162.31 (127.03 to 197.58) | 91.32 (79.70 to 102.93) | 261.97 (238.00 to 285.95) | 0.89 (-5.55 to 7.33) | **170.66 (137.03 to 204.28)** | **157.76 (121.20 to 194.32)** |
| Sadness and despair | | | | | | | | | | |
| No | 103.89 (94.23 to 113.54) | 112.01 (100.82 to 123.20) | 80.11 (67.91 to 92.32) | 78.82 (66.40 to 91.24) | 143.69 (119.03 to 168.35) | 121.03 (111.22 to 130.84) | 254.02 (231.52 to 276.53) | **5.95 (2.35 to 9.56)** | **132.99 (108.37 to 157.62)** | **117.90 (86.96 to 148.84)** |
| Yes | 117.96 (105.51 to 130.41) | 111.28 (98.59 to 123.96) | 110.29 (92.55 to 128.02) | 88.79 (75.33 to 102.24) | 162.92 (141.17 to 184.66) | 125.65 (109.46 to 141.84) | 264.34 (239.73 to 288.95) | **4.51 (0.00 to 9.03)** | **138.69 (106.48 to 170.90)** | **124.53 (87.79 to 161.28)** |
| Highest educational level of parents | | | | | | | | | | |
| High school or lower | 104.36 (95.17 to 113.54) | 104.00 (90.87 to 117.13) | 80.48 (67.87 to 93.09) | 81.77 (71.91 to 91.62) | 126.78 (102.52 to 151.03) | 142.43 (123.71 to 161.15) | 248.18 (214.77 to 281.58) | **7.96 (2.98 to 12.94)** | **105.75 (57.77 to 153.72)** | **89.59 (35.33 to 143.86)** |
| College or higher | 115.82 (103.46 to 128.17) | 116.53 (102.52 to 130.53) | 102.31 (89.47 to 115.15) | 83.84 (67.37 to 100.31) | 173.07 (151.97 to 194.17) | 116.79 (106.75 to 126.83) | 259.34 (239.71 to 278.97) | 3.03 (-0.55 to 6.60) | **142.55 (120.73 to 164.37)** | **128.54 (103.18 to 153.91)** |

Abbreviations: BMI, body mass index (calculated as weight in kilograms divided by height in meters squared); CI, confidence interval; KYRBS, Korea Youth Risk Behavior Web-Based Survey.

Numbers in bold indicate a significant difference (*P* < 0.05).

^a^ BMI was divided into four groups according to the 2017 Korean National Growth Charts: underweight (<5 percentile), normal (5 to 84 percentile), overweight (85 to 94 percentile), and obese (≥95 percentile).

^b^ School performance was divided into five groups: low (<20 percentile), middle-low (20 to 39 percentile), middle (40 to 59 percentile), middle-high (60 to 79 percentile), and high (≥80 percentile).

^c^ Physical activity frequency was divided into the three groups based on the engagement in vigorous aerobic and resistance trainings more than three days per week: lower activity (neither activity is done for more than three days per week), moderate activity (either one activity), and higher activity (both activities).

**Table S4.** The trend in the average screen time (m/d) of adolescents in orphanage group before and during COVID-19 pandemic, weighted mean (95% CI), in KYRBS.

|  | Pre-pandemic | | | | | | Pandemic | Trends in the pre-pandemic, β (95% CI) | Trends in the pandemic, β　(95% CI) | Trend differences, β_diff_ (95% CI) |
| --- | --- | --- | --- | --- | --- | --- | --- | --- | --- | --- |
| Year | 2008-2009 | 2010-2011 | 2012-2013 | 2014-2015 | 2016-2017 | 2018-2019 | 2020-2022 |  |  |  |
| Overall | 104.86 (87.83 to 121.88) | 88.01 (67.44 to 108.59) | 97.18 (78.19 to 116.16) | 75.60 (61.26 to 89.93) | 230.13 (199.79 to 260.46) | 136.13 (118.92 to 153.33) | 370.65 (337.13 to 404.18) | **16.01 (10.21 to 21.80)** | **234.53 (189.22 to 279.83)** | **221.90 (159.62 to 284.17)** |
| Sex | | | | | | | | | | |
| Male | 107.17 (86.30 to 128.04) | 97.14 (74.94 to 119.33) | 83.71 (64.67 to 102.75) | 81.29 (65.00 to 97.58) | 208.13 (181.26 to 234.99) | 130.19 (114.47 to 145.91) | 318.86 (281.13 to 356.59) | 12.33 (4.77 to 19.89) | 188.67 (128.16 to 249.19) | 178.21 (106.23 to 250.19) |
| Female | 100.29 (71.93 to 128.65) | 70.60 (53.74 to 87.47) | 114.20 (85.25 to 143.16) | 67.39 (43.97 to 90.80) | 264.85 (222.33 to 307.36) | 145.73 (114.53 to 176.92) | 430.78 (389.06 to 472.49) | 22.27 (12.02 to 32.53) | 285.05 (213.46 to 356.64) | 265.38 (169.53 to 361.24) |
| BMI group ^a^ | | | | | | | | | | |
| Underweight | 93.27 | 60.92 (39.54 to 82.31) | 96.8 | 60.22 (43.97 to 76.48) | 138.36 (115.82 to 160.90) | 76.92 (54.98 to 98.86) | 291.60 (59.12 to 524.08) | 1.83 (-9.14 to 12.80) | 214.68 (44.73 to 384.63) | 210.74 (38.95 to 382.53) |
| Normal | 100.36 (80.23 to 120.48) | 82.62 (68.63 to 96.61) | 52.34 (43.07 to 61.61) | 58.63 (44.73 to 72.52) | 182.34 (158.92 to 205.76) | 127.56 (113.06 to 142.07) | 357.11 (327.04 to 387.19) | 11.46 (4.77 to 18.15) | 229.55 (177.10 to 282.01) | 215.97 (152.59 to 279.35) |
| Overweight | 71.45 | 89.36 | 91.37 (90.40 to 92.34) | 67.61 | 335.48 (54.44 to 616.51) | 174.70 (131.98 to 217.43) | 441.53 (186.83 to 696.23) | 33.13 (5.74 to 60.53) | 266.82 (11.78 to 521.86) | 241.63 (-39.65 to 522.92) |
| Obese | 102.82 (78.43 to 127.21) | 71.92 | 115.58 | 85.07 | 260.97 (212.32 to 309.61) | 102.63 (92.16 to 113.10) | 341.94 (288.21 to 395.67) | 19.04 (-3.23 to 41.32) | 239.31 (112.84 to 365.78) | 217.12 (66.94 to 367.29) |
| Grade | | | | | | | | | | |
| 7^th^ | 97.30 (59.58 to 135.01) | 67.33 (52.50 to 82.16) | 120.94 (67.18 to 174.70) | 60.75 (43.59 to 77.91) | 157.43 (71.47 to 243.39) | 156.72 (108.53 to 204.91) | 253.91 (164.91 to 342.91) | 10.39 (-5.32 to 26.11) | 97.19 (-23.57 to 217.95) | 91.19 (-39.96 to 222.34) |
| 8^th^ | 122.66 (81.79 to 163.53) | 71.78 (45.67 to 97.89) | 86.51 (69.55 to 103.46) | 100.57 (69.71 to 131.44) | 202.83 (141.27 to 264.40) | 112.87 (70.20 to 155.54) | 412.42 (316.79 to 508.06) | 8.65 (-4.74 to 22.03) | 299.56 (179.96 to 419.15) | 287.81 (160.66 to 414.96) |
| 9^th^ | 105.82 (69.75 to 141.88) | 138.75 (82.99 to 194.52) | 113.04 (82.71 to 143.37) | 94.54 (61.03 to 128.05) | 210.21 (127.75 to 292.68) | 139.32 (113.89 to 164.74) | 396.64 (335.92 to 457.37) | 10.37 (-4.86 to 25.60) | 257.33 (141.00 to 373.65) | 243.69 (117.01 to 370.37) |
| 10^th^ | 88.06 (79.16 to 96.97) | 93.67 (71.84 to 115.50) | 71.09 (31.94 to 110.25) | 39.49 (33.63 to 45.35) | 285.51 (248.99 to 322.03) | 173.59 (124.96 to 222.22) | 378.12 (347.36 to 408.89) | 30.44 (16.78 to 44.10) | 204.53 (82.39 to 326.67) | 186.31 (36.68 to 335.94) |
| 11^th^ | 88.89 (78.05 to 99.72) | 83.43 (22.96 to 143.90) | 108.33 (80.20 to 136.47) | 86.96 (48.99 to 124.93) | 212.72 (177.62 to 247.82) | 127.65 (107.25 to 148.05) | 377.30 (305.07 to 449.54) | 16.54 (3.32 to 29.76) | 249.65 (136.38 to 362.93) | 235.17 (107.76 to 362.58) |
| 12^th^ | 122.95 (91.19 to 154.71) | 48.64 (44.04 to 53.24) | 79.52 (48.39 to 110.66) | 47.06 (30.16 to 63.95) | 255.26 (174.93 to 335.59) | 128.97 (97.38 to 160.56) | 357.58 (309.56 to 405.60) | 17.42 (2.39 to 32.46) | 228.61 (137.92 to 319.30) | 212.93 (102.15 to 323.70) |
| Region of residence | | | | | | | | | | |
| Urban | 115.66 (91.32 to 140.00) | 84.16 (56.55 to 111.78) | 99.70 (74.93 to 124.47) | 74.18 (55.38 to 92.98) | 227.26 (187.17 to 267.36) | 132.41 (106.04 to 158.78) | 370.94 (326.47 to 415.41) | 14.40 (6.69 to 22.12) | 238.53 (179.23 to 297.83) | 225.67 (152.22 to 299.13) |
| Rural | 86.86 (66.37 to 107.36) | 95.68 (67.00 to 124.37) | 93.52 (62.96 to 124.08) | 78.26 (56.18 to 100.34) | 235.81 (190.88 to 280.73) | 142.01 (125.10 to 158.92) | 370.06 (322.09 to 418.04) | 18.76 (10.22 to 27.29) | 228.05 (159.34 to 296.77) | 211.84 (124.76 to 298.92) |
| Smoking status | | | | | | | | | | |
| No | 81.25 (65.27 to 97.23) | 75.26 (63.65 to 86.86) | 59.64 (48.80 to 70.47) | 72.31 (55.98 to 88.64) | 207.86 (180.54 to 235.18) | 109.39 (93.96 to 124.82) | 366.02 (332.36 to 399.69) | 16.31 (10.54 to 22.08) | 256.63 (211.08 to 302.19) | 241.06 (178.23 to 303.89) |
| Yes | 140.59 (109.46 to 171.71) | 117.82 (74.14 to 161.50) | 235.02 (197.70 to 272.33) | 85.54 (64.70 to 106.37) | 295.34 (247.23 to 343.46) | 238.48 (185.68 to 291.28) | 398.94 (321.82 to 476.06) | 23.99 (8.12 to 39.86) | 160.46 (-4.98 to 325.91) | 145.18 (-37.78 to 328.14) |
| Alcoholic consumption | | | | | | | | | | |
| No | 83.35 (69.82 to 96.88) | 75.00 (62.69 to 87.31) | 64.30 (48.23 to 80.38) | 73.97 (57.20 to 90.75) | 186.49 (158.73 to 214.25) | 113.33 (98.95 to 127.70) | 339.75 (308.40 to 371.11) | 14.20 (8.29 to 20.11) | 226.42 (177.15 to 275.70) | 212.41 (149.30 to 275.51) |
| Yes | 136.99 (102.79 to 171.18) | 108.64 (73.06 to 144.22) | 142.87 (119.92 to 165.82) | 78.46 (60.34 to 96.59) | 301.25 (252.03 to 350.46) | 170.49 (140.07 to 200.92) | 436.89 (393.93 to 479.86) | 19.19 (6.95 to 31.43) | 266.40 (168.89 to 363.91) | 251.43 (136.10 to 366.77) |
| School performance ^b^ | | | | | | | | | | |
| Low | 84.75 (65.74 to 103.76) | 84.44 (58.22 to 110.67) | 98.89 (72.43 to 125.36) | 86.23 (63.84 to 108.62) | 296.18 (235.35 to 357.02) | 156.59 (128.69 to 184.50) | 441.51 (388.94 to 494.07) | 27.97 (17.84 to 38.11) | 284.92 (194.42 to 375.41) | 265.73 (148.39 to 383.06) |
| Middle-low | 132.44 (109.95 to 154.93) | 73.27 (60.06 to 86.49) | 50.97 (36.74 to 65.20) | 60.90 (55.70 to 66.10) | 174.55 (145.18 to 203.93) | 139.61 (109.56 to 169.65) | 343.87 (305.03 to 382.71) | 7.32 (-3.02 to 17.66) | 204.26 (130.72 to 277.81) | 191.73 (110.45 to 273.02) |
| Middle | 82.97 (52.74 to 113.20) | 72.43 (65.21 to 79.65) | 73.83 (32.22 to 115.43) | 56.46 (41.85 to 71.08) | 180.79 (164.67 to 196.91) | 144.37 (95.86 to 192.89) | 255.04 (211.30 to 298.77) | 19.19 (6.62 to 31.76) | 110.67 (30.59 to 190.75) | 97.29 (-4.14 to 198.73) |
| Middle-high | 66.02 (58.78 to 73.26) | 64.73 | 68.55 (-29.94 to 167.03) | 54.78 (44.85 to 64.70) | 118.37 (103.80 to 132.94) | 96.48 (78.08 to 114.89) | 273.54 (211.43 to 335.64) | 7.87 (-1.45 to 17.20) | 177.05 (83.45 to 270.66) | 166.40 (66.91 to 265.89) |
| High | 170.99 (100.12 to 241.87) | 158.89 (70.87 to 246.91) | 155.10 (127.91 to 182.29) | 87.69 (78.32 to 97.06) | 223.99 (178.91 to 269.06) | 124.11 (80.16 to 168.06) | 407.07 (337.50 to 476.63) | -2.31 (-19.88 to 15.25) | 282.96 (159.43 to 406.49) | 275.74 (148.65 to 402.83) |
| Sexual experience | | | | | | | | | | |
| No | 100.38 (78.91 to 121.84) | 71.09 (59.83 to 82.35) | 48.73 (39.47 to 57.99) | 67.02 (54.27 to 79.77) | 169.81 (144.54 to 195.08) | 123.43 (108.20 to 138.65) | 347.18 (315.44 to 378.91) | 12.29 (5.75 to 18.83) | 223.75 (175.32 to 272.19) | 210.70 (149.81 to 271.60) |
| Yes | 109.13 (79.68 to 138.57) | 103.55 (75.60 to 131.49) | 152.70 (133.04 to 172.36) | 86.47 (65.28 to 107.66) | 308.61 (273.79 to 343.44) | 151.44 (125.98 to 176.91) | 410.68 (351.90 to 469.46) | 21.99 (11.24 to 32.74) | 259.24 (162.38 to 356.10) | 242.39 (126.19 to 358.58) |
| Physical activity frequency ^c^ | | | | | | | | | | |
| Lower activity | 110.13 (86.27 to 133.99) | 97.91 (69.29 to 126.53) | 102.46 (84.62 to 120.29) | 84.96 (63.74 to 106.19) | 251.49 (204.09 to 298.89) | 145.89 (126.60 to 165.17) | 384.23 (339.61 to 428.86) | 17.48 (8.43 to 26.53) | 238.35 (175.09 to 301.61) | 222.88 (140.99 to 304.77) |
| Moderate activity | 94.57 (70.53 to 118.61) | 67.86 (47.72 to 87.99) | 62.58 (45.61 to 79.55) | 58.57 (33.29 to 83.86) | 160.53 (146.51 to 174.56) | 123.67 (92.96 to 154.38) | 317.78 (243.78 to 391.78) | 11.81 (2.69 to 20.92) | 194.11 (79.30 to 308.92) | 182.44 (60.53 to 304.36) |
| Higher activity | 106.32 (87.46 to 125.18) | 94.85 (79.26 to 110.43) | 133.00 (100.91 to 165.08) | 80.02 (58.95 to 101.10) | 284.78 (237.25 to 332.31) | 130.07 (98.72 to 161.42) | 372.38 (313.20 to 431.56) | 17.42 (4.38 to 30.46) | 242.31 (134.74 to 349.87) | 229.41 (106.39 to 352.43) |
| Sadness and despair | | | | | | | | | | |
| No | 84.66 (61.01 to 108.31) | 76.88 (59.68 to 94.07) | 64.40 (48.17 to 80.63) | 68.88 (53.82 to 83.95) | 190.91 (169.24 to 212.57) | 124.91 (109.40 to 140.42) | 355.87 (316.15 to 395.58) | 17.11 (10.52 to 23.70) | 230.96 (172.29 to 289.62) | 215.87 (141.05 to 290.69) |
| Yes | 121.30 (101.92 to 140.68) | 98.76 (71.90 to 125.63) | 131.21 (109.04 to 153.38) | 84.38 (64.32 to 104.45) | 304.96 (249.09 to 360.82) | 149.71 (114.39 to 185.03) | 391.08 (344.55 to 437.61) | 17.46 (6.48 to 28.45) | 241.37 (165.48 to 317.25) | 227.21 (133.04 to 321.38) |
| Highest educational level of parents | | | | | | | | | | |
| High school or lower | 91.3 | 89.06 (1.84 to 176.27) | 86.83 | 124.33 | 283.37 | 132.6 | 409.62 | 25.89 (-4.27 to 56.06) | 277.02 (72.59 to 481.46) | 260.87 (28.78 to 492.96) |
| College or higher | 105.83 (87.93 to 123.74) | 87.94 (66.08 to 109.81) | 97.65 (77.74 to 117.57) | 73.64 (59.10 to 88.18) | 227.74 (198.24 to 257.23) | 136.25 (118.80 to 153.69) | 368.90 (335.98 to 401.83) | 15.60 (9.63 to 21.57) | 232.66 (186.20 to 279.12) | 218.65 (155.84 to 281.46) |

Abbreviations: BMI, body mass index (calculated as weight in kilograms divided by height in meters squared); CI, confidence interval; KYRBS, Korea Youth Risk Behavior Web-Based Survey.

Numbers in bold indicate a significant difference (*P* < 0.05).

^a^ BMI was divided into four groups according to the 2017 Korean National Growth Charts: underweight (<5 percentile), normal (5 to 84 percentile), overweight (85 to 94 percentile), and obese (≥95 percentile).

^b^ School performance was divided into five groups: low (<20 percentile), middle-low (20 to 39 percentile), middle (40 to 59 percentile), middle-high (60 to 79 percentile), and high (≥80 percentile).

^c^ Physical activity frequency was divided into the three groups based on the engagement in vigorous aerobic and resistance trainings more than three days per week: lower activity (neither activity is done for more than three days per week), moderate activity (either one activity), and higher activity (both activities).**Table S5.** Factors associated with weighted average screen time among adolescents in nuclear family group before and during COVID-19 pandemic in KYRBS.

| Factors | Unadjusted model | | | Adjusted model ^a^ | | |
| --- | --- | --- | --- | --- | --- | --- |
|  | Pre-pandemic screen time, β (95% CI) | Pandemic screen time, β (95% CI) | β_diff_ (95% CI) | Pre-pandemic screen time, β (95% CI) | Pandemic screen time, β (95% CI) | β_diff_ (95% CI) |
| **Biological factors** | | | | | | |
| Sex | | | | | | |
| Male | 1.00 (ref) | 1.00 (ref) | 1.00 (ref) | 1.00 (ref) | 1.00 (ref) | 1.00 (ref) |
| Female | **-6.22 (-7.70 to -4.74)** | **63.46 (59.51 to 67.42)** | **69.69 (65.47 to 73.91)** | **-10.50 (-11.92 to -9.08)** | **63.65 (59.98 to 67.31)** | **74.14 (70.21 to 78.07)** |
| BMI group ^b^ | | | | | | |
| Underweight | 1.00 (ref) | 1.00 (ref) | 1.00 (ref) | 1.00 (ref) | 1.00 (ref) | 1.00 (ref) |
| Obese | **5.13 (4.47 to 5.79)** | 0.51 (-1.45 to 2.47) | **-4.62 (-6.69 to -2.55)** | **3.48 (2.83 to 4.14)** | **2.74 (0.91 to 4.57)** | -0.74 (-2.68 to 1.21) |
| **Social factors** | | | | | | |
| Grade | | | | | | |
| 7^th^ | 1.00 (ref) | 1.00 (ref) | 1.00 (ref) | 1.00 (ref) | 1.00 (ref) | 1.00 (ref) |
| 12^th^ | -0.24 (-0.60 to 0.12) | **11.69 (10.33 to 13.06)** | **11.94 (10.53 to 13.35)** | **-5.63 (-6.54 to -4.72)** | **3.72 (0.32 to 7.11)** | **9.34 (5.83 to 12.86)** |
| Region of residence | | | | | | |
| Rural | 1.00 (ref) | 1.00 (ref) | 1.00 (ref) | 1.00 (ref) | 1.00 (ref) | 1.00 (ref) |
| Urban | **-1.59 (-3.07 to -0.11)** | **-14.10 (-19.42 to -8.78)** | **-12.51 (-18.03 to -6.99)** | 0.70 (-0.70 to 2.10) | **-7.50 (-11.59 to -3.41)** | **-8.20 (-12.53 to -3.88)** |
| Smoking status | | | | | | |
| No | 1.00 (ref) | 1.00 (ref) | 1.00 (ref) | 1.00 (ref) | 1.00 (ref) | 1.00 (ref) |
| Yes | **9.43 (7.16 to 11.70)** | **192.44 (171.31 to 213.57)** | **183.01 (161.75 to 204.26)** | **5.71 (3.26 to 8.16)** | **97.22 (76.44 to 118.01)** | **91.52 (70.59 to 112.44)** |
| Alcoholic consumption | | | | | | |
| No | 1.00 (ref) | 1.00 (ref) | 1.00 (ref) | 1.00 (ref) | 1.00 (ref) | 1.00 (ref) |
| Yes | **14.03 (12.74 to 15.32)** | **96.89 (91.01 to 102.77)** | **82.86 (76.84 to 88.89)** | **12.71 (11.34 to 14.07)** | **67.07 (61.58 to 72.57)** | **54.37 (48.70 to 60.03)** |
| School performance ^c^ | | | | | | |
| Low | 1.00 (ref) | 1.00 (ref) | 1.00 (ref) | 1.00 (ref) | 1.00 (ref) | 1.00 (ref) |
| High | **10.08 (9.65 to 10.51)** | **43.45 (42.05 to 44.84)** | **33.37 (31.91 to 34.83)** | **8.87 (8.44 to 9.30)** | **34.15 (32.78 to 35.51)** | **25.28 (23.85 to 26.70)** |
| Sexual experience | | | | | | |
| No | 1.00 (ref) | 1.00 (ref) | 1.00 (ref) | 1.00 (ref) | 1.00 (ref) | 1.00 (ref) |
| Yes | 0.47 (-1.09 to 2.02) | **97.83 (88.38 to 107.27)** | **97.36 (87.79 to 106.94)** | 1.23 (-0.39 to 2.86) | **53.75 (44.97 to 62.54)** | **52.52 (43.59 to 61.46)** |
| Physical activity frequency ^d^ | | | | | | |
| Lower activity | 1.00 (ref) | 1.00 (ref) | 1.00 (ref) | 1.00 (ref) | 1.00 (ref) | 1.00 (ref) |
| Higher activity | **-3.78 (-4.45 to -3.11)** | **-14.49 (-16.54 to -12.43)** | **-10.71 (-12.87 to -8.54)** | **-7.23 (-7.87 to -6.59)** | **-3.43 (-5.37 to -1.48)** | **3.81 (1.76 to 5.86)** |
| Sadness and despair | | | | | | |
| No | 1.00 (ref) | 1.00 (ref) | 1.00 (ref) | 1.00 (ref) | 1.00 (ref) | 1.00 (ref) |
| Yes | **5.92 (4.95 to 6.88)** | **44.98 (41.39 to 48.58)** | **39.07 (35.35 to 42.79)** | **7.39 (6.41 to 8.37)** | **20.73 (17.34 to 24.13)** | **13.34 (9.81 to 16.88)** |
| **Familial factor** | | | | | | |
| Highest education level of parents | | | | | | |
| High school or lower | 1.00 (ref) | 1.00 (ref) | 1.00 (ref) | 1.00 (ref) | 1.00 (ref) | 1.00 (ref) |
| College or higher | **-15.33 (-16.30 to -14.36)** | **-67.98 (-71.56 to -64.40)** | **-52.651 (-56.36 to -48.94)** | **-13.71 (-14.68 to -12.73)** | **-46.85 (-50.29 to -43.41)** | **-33.15 (-36.72 to -29.57)** |

Abbreviations: BMI, body mass index (calculated as weight in kilograms divided by height in meters squared); CI, confidence interval; KYRBS, Korea Youth Risk Behavior Web-Based Survey.

Numbers in bold indicate a significant difference (*P* < 0.05).

^a^ Adjustment for age, sex, BMI group, grade, region of residence, smoking status, alcoholic consumption, school performance, sexual experience, physical activity frequency, sadness and despair, and highest education level of parents.

^b^ BMI was divided into four groups according to the 2017 Korean National Growth Charts: underweight (<5 percentile), normal (5 to 84 percentile), overweight (85 to 94 percentile), and obese (≥95 percentile).

^c^ School performance was divided into five groups: low (<20 percentile), middle-low (20 to 39 percentile), middle (40 to 59 percentile), middle-high (60 to 79 percentile), and high (≥80 percentile).

^d^ Physical activity frequency was divided into the three groups based on the engagement in vigorous aerobic and resistance trainings more than three days per week: lower activity (neither activity is done for more than three days per week), moderate activity (either one activity), and higher activity (both activities).

**Table S6.** Factors associated with weighted average screen time among adolescents in living with relatives group before and during COVID-19 pandemic in KYRBS.

| Factors | Unadjusted model | | | Adjusted model ^a^ | | |
| --- | --- | --- | --- | --- | --- | --- |
|  | Pre-pandemic screen time, β (95% CI) | Pandemic screen time, β (95% CI) | β_diff_ (95% CI) | Pre-pandemic screen time, β (95% CI) | Pandemic screen time, β (95% CI) | β_diff_ (95% CI) |
| **Biological factors** | | | | | | |
| Sex | | | | | | |
| Male | 1.00 (ref) | 1.00 (ref) | 1.00 (ref) | 1.00 (ref) | 1.00 (ref) | 1.00 (ref) |
| Female | **-8.98 (-15.14 to -2.82)** | **109.58 (84.72 to 134.44)** | **118.56 (92.95 to 144.17)** | **-15.14 (-21.58 to -8.71)** | **102.65 (76.93 to 128.36)** | **117.79 (91.28 to 144.30)** |
| BMI group ^b^ | | | | | | |
| Underweight | 1.00 (ref) | 1.00 (ref) | 1.00 (ref) | 1.00 (ref) | 1.00 (ref) | 1.00 (ref) |
| Obese | **13.00 (9.46 to 16.54)** | **14.42 (1.27 to 27.57)** | 1.42 (-12.20 to 15.04) | **11.43 (7.85 to 15.02)** | **22.32 (9.90 to 34.74)** | 10.89 (-2.04 to 23.81) |
| **Social factors** | | | | | | |
| Grade | | | | | | |
| 7^th^ | 1.00 (ref) | 1.00 (ref) | 1.00 (ref) | 1.00 (ref) | 1.00 (ref) | 1.00 (ref) |
| 12^th^ | **2.34 (0.57 to 4.12)** | 3.53 (-3.84 to 10.89) | 1.18 (-6.40 to 8.76) | 0.62 (-4.48 to 5.73) | -8.84 (-31.12 to 13.44) | -9.47 (-32.32 to 13.39) |
| Region of residence | | | | | | |
| Rural | 1.00 (ref) | 1.00 (ref) | 1.00 (ref) | 1.00 (ref) | 1.00 (ref) | 1.00 (ref) |
| Urban | -3.66 (-9.67 to 2.35) | -11.63 (-39.14 to 15.88) | -7.97 (-36.13 to 20.19) | -2.75 (-8.72 to 3.22) | -12.26 (-37.15 to 12.62) | -9.51 (-35.10 to 16.08) |
| Smoking status | | | | | | |
| No | 1.00 (ref) | 1.00 (ref) | 1.00 (ref) | 1.00 (ref) | 1.00 (ref) | 1.00 (ref) |
| Yes | -9.01 (-18.22 to 0.20) | 53.50 (-89.09 to 196.09) | 62.51 (-80.38 to 205.39) | -1.02 (-10.96 to 8.93) | 15.21 (-131.56 to 161.98) | 16.23 (-130.88 to 163.33) |
| Alcoholic consumption | | | | | | |
| No | 1.00 (ref) | 1.00 (ref) | 1.00 (ref) | 1.00 (ref) | 1.00 (ref) | 1.00 (ref) |
| Yes | 1.43 (-5.43 to 8.28) | **57.42 (20.94 to 93.91)** | **56.00 (18.87 to 93.12)** | 3.68 (-3.82 to 11.17) | **40.99 (6.19 to 75.79)** | **37.31 (1.71 to 72.91)** |
| School performance ^c^ | | | | | | |
| Low | 1.00 (ref) | 1.00 (ref) | 1.00 (ref) | 1.00 (ref) | 1.00 (ref) | 1.00 (ref) |
| High | **6.67 (4.20 to 9.15)** | **61.64 (51.04 to 72.25)** | **54.97 (44.08 to 65.86)** | **7.76 (5.26 to 10.27)** | **61.20 (50.54 to 71.86)** | **53.44 (42.48 to 64.39)** |
| Sexual experience | | | | | | |
| No | 1.00 (ref) | 1.00 (ref) | 1.00 (ref) | 1.00 (ref) | 1.00 (ref) | 1.00 (ref) |
| Yes | **-8.85 (-16.43 to -1.27)** | 42.04 (-7.95 to 92.04) | **50.89 (0.33 to 101.46)** | -2.60 (-11.21 to 6.02) | **47.25 (2.79 to 91.71)** | **49.84 (4.55 to 95.13)** |
| Physical activity frequency ^d^ | | | | | | |
| Lower activity | 1.00 (ref) | 1.00 (ref) | 1.00 (ref) | 1.00 (ref) | 1.00 (ref) | 1.00 (ref) |
| Higher activity | **-7.45 (-11.30 to -3.61)** | **-22.70 (-39.96 to -5.44)** | -15.25 (-32.93 to 2.43) | **-9.89 (-13.89 to -5.89)** | -6.98 (-24.54 to 10.58) | 2.91 (-15.10 to 20.92) |
| Sadness and despair | | | | | | |
| No | 1.00 (ref) | 1.00 (ref) | 1.00 (ref) | 1.00 (ref) | 1.00 (ref) | 1.00 (ref) |
| Yes | -2.07 (-8.27 to 4.13) | **36.01 (8.14 to 63.87)** | **38.08 (9.54 to 66.62)** | 2.55 (-3.85 to 8.95) | -0.77 (-27.46 to 25.91) | -3.32 (-30.77 to 24.12) |
| **Familial factor** | | | | | | |
| Highest education level of parents | | | | | | |
| High school or lower | 1.00 (ref) | 1.00 (ref) | 1.00 (ref) | 1.00 (ref) | 1.00 (ref) | 1.00 (ref) |
| College or higher | 6.82 (0.00 to 13.64) | -6.69 (-34.35 to 20.97) | -13.51 (-42.00 to 14.98) | **7.92 (1.06 to 14.79)** | -1.58 (-27.36 to 24.20) | -9.50 (-36.18 to 17.18) |

Abbreviations: BMI, body mass index (calculated as weight in kilograms divided by height in meters squared); CI, confidence interval; KYRBS, Korea Youth Risk Behavior Web-Based Survey.

Numbers in bold indicate a significant difference (*P* < 0.05).

^a^ Adjustment for age, sex, BMI group, grade, region of residence, smoking status, alcoholic consumption, school performance, sexual experience, physical activity frequency, sadness and despair, and highest education level of parents.

^b^ BMI was divided into four groups according to the 2017 Korean National Growth Charts: underweight (<5 percentile), normal (5 to 84 percentile), overweight (85 to 94 percentile), and obese (≥95 percentile).

^c^ School performance was divided into five groups: low (<20 percentile), middle-low (20 to 39 percentile), middle (40 to 59 percentile), middle-high (60 to 79 percentile), and high (≥80 percentile).

^d^ Physical activity frequency was divided into the three groups based on the engagement in vigorous aerobic and resistance trainings more than three days per week: lower activity (neither activity is done for more than three days per week), moderate activity (either one activity), and higher activity (both activities).

**Table S7.** Factors associated with weighted average screen time among adolescents in living alone group before and during COVID-19 pandemic in KYRBS.

| Factors | Unadjusted model | | | Adjusted model ^a^ | | |
| --- | --- | --- | --- | --- | --- | --- |
|  | Pre-pandemic screen time, β (95% CI) | Pandemic screen time, β (95% CI) | β_diff_ (95% CI) | Pre-pandemic screen time, β (95% CI) | Pandemic screen time, β (95% CI) | β_diff_ (95% CI) |
| **Biological factors** | | | | | | |
| Sex | | | | | | |
| Male | 1.00 (ref) | 1.00 (ref) | 1.00 (ref) | 1.00 (ref) | 1.00 (ref) | 1.00 (ref) |
| Female | **17.91 (7.12 to 28.69)** | **54.34 (23.27 to 85.40)** | **36.43 (3.55 to 69.32)** | **14.80 (3.57 to 26.03)** | **55.60 (26.67 to 84.52)** | **40.80 (9.77 to 71.83)** |
| BMI group ^b^ | | | | | | |
| Underweight | 1.00 (ref) | 1.00 (ref) | 1.00 (ref) | 1.00 (ref) | 1.00 (ref) | 1.00 (ref) |
| Obese | **7.14 (0.22 to 14.06)** | **15.90 (1.06 to 30.74)** | 8.76 (-7.62 to 25.14) | **9.01 (2.34 to 15.68)** | **17.73 (4.46 to 30.99)** | 8.72 (-6.13 to 23.57) |
| **Social factors** | | | | | | |
| Grade | | | | | | |
| 7^th^ | 1.00 (ref) | 1.00 (ref) | 1.00 (ref) | 1.00 (ref) | 1.00 (ref) | 1.00 (ref) |
| 12^th^ | 3.83 (-0.61 to 8.28) | 0.68 (-10.00 to 11.36) | -3.15 (-14.72 to 8.42) | -0.76 (-8.35 to 6.82) | -11.26 (-32.37 to 9.86) | -10.49 (-32.93 to 11.94) |
| Region of residence | | | | | | |
| Rural | 1.00 (ref) | 1.00 (ref) | 1.00 (ref) | 1.00 (ref) | 1.00 (ref) | 1.00 (ref) |
| Urban | **13.73 (1.95 to 25.51)** | -4.04 (-47.07 to 38.99) | -17.77 (-62.38 to 26.85) | **12.27 (0.81 to 23.74)** | -7.17 (-44.61 to 30.27) | -19.44 (-58.60 to 19.72) |
| Smoking status | | | | | | |
| No | 1.00 (ref) | 1.00 (ref) | 1.00 (ref) | 1.00 (ref) | 1.00 (ref) | 1.00 (ref) |
| Yes | 16.79 (-4.24 to 37.81) | **212.31 (94.42 to 330.21)** | **195.53 (75.77 to 315.28)** | 8.47 (-13.61 to 30.55) | **164.67 (49.18 to 280.16)** | **156.20 (38.62 to 273.78)** |
| Alcoholic consumption | | | | | | |
| No | 1.00 (ref) | 1.00 (ref) | 1.00 (ref) | 1.00 (ref) | 1.00 (ref) | 1.00 (ref) |
| Yes | **36.96 (25.29 to 48.63)** | **79.36 (47.21 to 111.52)** | **42.40 (8.20 to 76.61)** | **37.07 (24.78 to 49.36)** | **53.08 (26.44 to 79.71)** | 16.01 (-13.33 to 45.34) |
| School performance ^c^ | | | | | | |
| Low | 1.00 (ref) | 1.00 (ref) | 1.00 (ref) | 1.00 (ref) | 1.00 (ref) | 1.00 (ref) |
| High | **8.61 (4.07 to 13.15)** | **41.63 (31.85 to 51.41)** | **33.02 (22.24 to 43.80)** | **6.93 (2.30 to 11.57)** | **38.09 (28.83 to 47.34)** | **31.16 (20.80 to 41.51)** |
| Sexual experience | | | | | | |
| No | 1.00 (ref) | 1.00 (ref) | 1.00 (ref) | 1.00 (ref) | 1.00 (ref) | 1.00 (ref) |
| Yes | 5.43 (-7.32 to 18.18) | **48.88 (8.88 to 88.87)** | **43.45 (1.47 to 85.43)** | -1.98 (-16.12 to 12.17) | 8.97 (-24.03 to 41.96) | 10.94 (-24.96 to 46.84) |
| Physical activity frequency ^d^ | | | | | | |
| Lower activity | 1.00 (ref) | 1.00 (ref) | 1.00 (ref) | 1.00 (ref) | 1.00 (ref) | 1.00 (ref) |
| Higher activity | **-8.49 (-15.11 to -1.86)** | -3.65 (-16.39 to 9.08) | 4.83 (-9.52 to 19.19) | **-7.73 (-14.71 to -0.76)** | -2.99 (-15.37 to 9.38) | 4.74 (-9.46 to 18.95) |
| Sadness and despair | | | | | | |
| No | 1.00 (ref) | 1.00 (ref) | 1.00 (ref) | 1.00 (ref) | 1.00 (ref) | 1.00 (ref) |
| Yes | 9.26 (-0.70 to 19.21) | 10.32 (-11.78 to 32.41) | 1.06 (-23.17 to 25.30) | 3.36 (-6.59 to 13.31) | -16.74 (-37.54 to 4.06) | -20.10 (-43.16 to 2.96) |
| **Familial factor** | | | | | | |
| Highest education level of parents | | | | | | |
| High school or lower | 1.00 (ref) | 1.00 (ref) | 1.00 (ref) | 1.00 (ref) | 1.00 (ref) | 1.00 (ref) |
| College or higher | **-11.17 (-21.79 to -0.55)** | -11.16 (-43.76 to 21.44) | 0.005 (-34.279 to 34.288) | -4.13 (-14.41 to 6.16) | -17.60 (-44.29 to 9.09) | -13.48 (-42.08 to 15.13) |

Abbreviations: BMI, body mass index (calculated as weight in kilograms divided by height in meters squared); CI, confidence interval; KYRBS, Korea Youth Risk Behavior Web-Based Survey.

Numbers in bold indicate a significant difference (*P* < 0.05).

^a^ Adjustment for age, sex, BMI group, grade, region of residence, smoking status, alcoholic consumption, school performance, sexual experience, physical activity frequency, sadness and despair, and highest education level of parents.

^b^ BMI was divided into four groups according to the 2017 Korean National Growth Charts: underweight (<5 percentile), normal (5 to 84 percentile), overweight (85 to 94 percentile), and obese (≥95 percentile).

^c^ School performance was divided into five groups: low (<20 percentile), middle-low (20 to 39 percentile), middle (40 to 59 percentile), middle-high (60 to 79 percentile), and high (≥80 percentile).

^d^ Physical activity frequency was divided into the three groups based on the engagement in vigorous aerobic and resistance trainings more than three days per week: lower activity (neither activity is done for more than three days per week), moderate activity (either one activity), and higher activity (both activities).

**Table S8.** Factors associated with weighted average screen time among adolescents in orphanage group before and during COVID-19 pandemic in KYRBS.

| Factors | Unadjusted model | | | Adjusted model ^a^ | | |
| --- | --- | --- | --- | --- | --- | --- |
|  | Pre-pandemic screen time, β (95% CI) | Pandemic screen time, β (95% CI) | β_diff_ (95% CI) | Pre-pandemic screen time, β (95% CI) | Pandemic screen time, β (95% CI) | β_diff_ (95% CI) |
| **Biological factors** | | | | | | |
| Sex | | | | | | |
| Male | 1.00 (ref) | 1.00 (ref) | 1.00 (ref) | 1.00 (ref) | 1.00 (ref) | 1.00 (ref) |
| Female | 8.04 (-13.39 to 29.47) | **111.92 (34.94 to 188.90)** | **103.88 (23.97 to 183.79)** | 3.55 (-17.76 to 24.87) | **109.05 (32.19 to 185.91)** | **105.50 (25.74 to 185.26)** |
| BMI group ^b^ | | | | | | |
| Underweight | 1.00 (ref) | 1.00 (ref) | 1.00 (ref) | 1.00 (ref) | 1.00 (ref) | 1.00 (ref) |
| Obese | **-14.48 (-24.11 to -4.85)** | -14.33 (-50.01 to 21.35) | 0.15 (-36.81 to 37.11) | -0.47 (-10.16 to 9.21) | -7.81 (-43.54 to 27.92) | -7.34 (-44.36 to 29.68) |
| **Social factors** | | | | | | |
| Grade | | | | | | |
| 7^th^ | 1.00 (ref) | 1.00 (ref) | 1.00 (ref) | 1.00 (ref) | 1.00 (ref) | 1.00 (ref) |
| 12^th^ | 0.73 (-5.35 to 6.80) | 1.25 (-21.68 to 24.19) | 0.53 (-23.20 to 24.25) | 3.36 (-10.03 to 16.75) | 12.42 (-37.57 to 62.42) | 9.06 (-42.69 to 60.82) |
| Region of residence | | | | | | |
| Rural | 1.00 (ref) | 1.00 (ref) | 1.00 (ref) | 1.00 (ref) | 1.00 (ref) | 1.00 (ref) |
| Urban | 2.46 (-17.63 to 22.55) | 0.88 (-77.69 to 79.44) | -1.59 (-82.68 to 79.51) | -0.30 (-19.38 to 18.77) | -8.15 (-88.03 to 71.72) | -7.85 (-89.97 to 74.27) |
| Smoking status | | | | | | |
| No | 1.00 (ref) | 1.00 (ref) | 1.00 (ref) | 1.00 (ref) | 1.00 (ref) | 1.00 (ref) |
| Yes | **72.81 (44.22 to 101.40)** | 32.92 (-115.55 to 181.38) | -39.89 (-191.09 to 111.30) | **59.06 (29.49 to 88.62)** | -67.46 (-232.18 to 97.26) | -126.51 (-293.87 to 40.84) |
| Alcoholic consumption | | | | | | |
| No | 1.00 (ref) | 1.00 (ref) | 1.00 (ref) | 1.00 (ref) | 1.00 (ref) | 1.00 (ref) |
| Yes | **55.49 (32.27 to 78.71)** | 97.14 (-1.72 to 196.00) | 41.65 (-59.90 to 143.20) | 20.55 (-2.66 to 43.77) | 106.49 (-10.86 to 223.83) | 85.94 (-33.69 to 205.56) |
| School performance ^c^ | | | | | | |
| Low | 1.00 (ref) | 1.00 (ref) | 1.00 (ref) | 1.00 (ref) | 1.00 (ref) | 1.00 (ref) |
| High | -3.60 (-11.00 to 3.80) | 12.62 (-19.56 to 44.80) | 16.22 (-16.80 to 49.24) | -1.37 (-8.62 to 5.88) | 22.34 (-9.75 to 54.43) | 23.71 (-9.19 to 56.61) |
| Sexual experience | | | | | | |
| No | 1.00 (ref) | 1.00 (ref) | 1.00 (ref) | 1.00 (ref) | 1.00 (ref) | 1.00 (ref) |
| Yes | **50.22 (29.03 to 71.40)** | 63.50 (-34.28 to 161.29) | 13.29 (-86.77 to 113.34) | 21.41 (-1.21 to 44.03) | 47.92 (-66.11 to 161.94) | 26.51 (-89.74 to 142.75) |
| Physical activity frequency ^d^ | | | | | | |
| Lower activity | 1.00 (ref) | 1.00 (ref) | 1.00 (ref) | 1.00 (ref) | 1.00 (ref) | 1.00 (ref) |
| Higher activity | -1.73 (-15.33 to 11.88) | -12.34 (-69.20 to 44.51) | -10.62 (-69.08 to 47.85) | -9.66 (-23.29 to 3.96) | -3.61 (-62.84 to 55.63) | 6.06 (-54.72 to 66.84) |
| Sadness and despair | | | | | | |
| No | 1.00 (ref) | 1.00 (ref) | 1.00 (ref) | 1.00 (ref) | 1.00 (ref) | 1.00 (ref) |
| Yes | **34.18 (12.21 to 56.16)** | 35.21 (-50.17 to 120.60) | 1.03 (-87.14 to 89.20) | 17.11 (-4.33 to 38.56) | 4.26 (-83.17 to 91.70) | -12.85 (-102.87 to 77.18) |
| **Familial factor** | | | | | | |
| Highest education level of parents | | | | | | |
| High school or lower | 1.00 (ref) | 1.00 (ref) | 1.00 (ref) | 1.00 (ref) | 1.00 (ref) | 1.00 (ref) |
| College or higher | 5.69 (-36.82 to 48.20) | 40.71 (-114.96 to 196.39) | 35.022 (-126.354 to 196.398) | 13.64 (-30.43 to 57.72) | 20.36 (-138.35 to 179.07) | 6.72 (-158.00 to 171.44) |

Abbreviations: BMI, body mass index (calculated as weight in kilograms divided by height in meters squared); CI, confidence interval; KYRBS, Korea Youth Risk Behavior Web-Based Survey.

Numbers in bold indicate a significant difference (*P* < 0.05).

^a^ Adjustment for age, sex, BMI group, grade, region of residence, smoking status, alcoholic consumption, school performance, sexual experience, physical activity frequency, sadness and despair, and highest education level of parents.

^b^ BMI was divided into four groups according to the 2017 Korean National Growth Charts: underweight (<5 percentile), normal (5 to 84 percentile), overweight (85 to 94 percentile), and obese (≥95 percentile).

^c^ School performance was divided into five groups: low (<20 percentile), middle-low (20 to 39 percentile), middle (40 to 59 percentile), middle-high (60 to 79 percentile), and high (≥80 percentile).

^d^ Physical activity frequency was divided into the three groups based on the engagement in vigorous aerobic and resistance trainings more than three days per week: lower activity (neither activity is done for more than three days per week), moderate activity (either one activity), and higher activity (both activities).
